# An economic evaluation of cattle tick acaricide-resistances and the financial losses in subtropical dairy farms of Ecuador: A farm system approach

**DOI:** 10.1371/journal.pone.0287104

**Published:** 2023-06-29

**Authors:** Valeria Paucar-Quishpe, Ximena Pérez-Otáñez, Richar Rodríguez-Hidalgo, Darío Cepeda-Bastidas, Cecilia Pérez-Escalante, Jorge Grijalva-Olmedo, Sandra Enríquez, Susana Arciniegas-Ortega, Luis Sandoval-Trávez, Bryan Benavides-Erazo, Sophie O. Vanwambeke, Claude Saegerman, Lenin Ron-Garrido

**Affiliations:** 1 Instituto de Investigación en Zoonosis (CIZ), Universidad Central del Ecuador, Quito, Ecuador; 2 Research Unit of Epidemiology and Risk Analysis applied to Veterinary Science (UREAR-ULg)/Fundamental and Applied Research for Animals & Health (FARAH) Center/Faculty of Veterinary Medicine, University of Liege, Liège, Belgium; 3 Georges Lemaitre Centre for Earth and Climate Research, Earth & Life Institute, UCLouvain, Louvain-la-Neuve, Belgium; 4 Facultad de Medicina Veterinaria y Zootecnia, Universidad Central del Ecuador, Quito, Ecuador; 5 Facultad de Ciencias Agrícolas, Universidad Central del Ecuador, Quito, Ecuador; 6 Facultad de Geología, Minas y Petróleo, Universidad Central del Ecuador, Quito, Ecuador; Cairo University Faculty of Veterinary Medicine, EGYPT

## Abstract

Estimates of economic losses in cattle due to tick infestations in subtropical areas are limited, such as in Ecuador. Ticks affect animal production and health, but those direct effects are difficult to estimate since financial exercises carried out in farms consider both costs of the inputs and revenues. This study aims to quantify the costs of inputs involved in milk production and to know the role of acaricide treatment in the production costs on dairy farms in subtropical zones using a farming system approach. Regression and classification trees were used to study the relationship between tick control, acaricide resistance and the presence of high level of tick infestation in the farm system. Even though there was no significant direct association between high levels of tick infestation and the presence of acaricide resistance in ticks, a more complex structure for resistances operates in the manifestation of high tick infestation involving levels of farm technology and no acaricide resistance. Farms with higher levels of technology allocate a lower percentage of sanitary expenses to control ticks (13.41%) in comparison to semi-technified (23.97%) and non-technified farms (32.49%). Likewise, more technified and bigger herds have a lower annual expenditure on acaricide treatment (1.30% of the production budget equivalent to 8.46 USD per animal) compared to non-technified farms where it can represent more than 2.74% of the production budget and where the absence of cypermethrin resistance increases the treatment cost to 19.50 USS per animal annually. These results can motivate the development of information campaigns and control programmes targeted to the reality of small and medium farms that are the most affected in terms of the money they invest in controlling ticks.

## Introduction

Ticks affect almost 80% of the world’s cattle population in tropical and subtropical areas. These ectoparasites cause impacts on animal production and health, either through the effect of their bites, which cause anaemia when high parasite loads are present or through the transmission of pathogens [1]. In addition, there are economic losses associated with control of tick infestations or tick-borne diseases’ (TBD) treatment; and the revenue not perceived due to reduced meat or milk production are part of the indirect costs [2, 3]. In Ecuador, approximately 6.15 million litres of raw milk are produced daily, according to the National Institute of Statistics (INEC) [4]. Of them, herds located in tropical and subtropical areas produce around 1.5 million litres of milk/day (25% of national production), and most of them belong to small size farms [4, 5]. Livestock production in those areas is very suitable for tick infestation because of the humid tropical climate, the use of specialised dairy breeds, and sowing of pastures of short-cycle as the primary feed source [6, 7].

On the other hand, nowadays, the main usual tool for tick control is the use of chemical acaricides. In Ecuador, the most commonly used acaricides for tick control are amitraz, ivermectin, and alpha-cypermethrin [8]. However, other acaricides based on organophosphates, fluazuron, fipronil, and other ingredients are also available in the market. There are also various forms of application and presentations of these products, such as fluid products or wet-powder that are diluted and sprayed on animals. Pour-on or injectable control methods are easy to use but are expensive or generate residues in milk and meat for several weeks after [9–11]. Likewise, incorrect use of these pesticides, such as under dosing, inadequate preparations, and misapplications, can lead to treatment failure. Added to this, the exclusive strategy of chemical control may be inadequate due to the development of tick resistance [12, 13]. Various levels of presence of resistance in *Rhipicephalus microplus* were observed to amitraz [14, 15], alpha-cypermethrin, and ivermectin [15] in some producing areas of the Ecuadorian Coast.

Estimations of the economic losses due to tick infestation, especially in tropical endemic areas, are scarce. The main strategies used to find the effects are obtained from specific experiments designed to evaluate tick load versus reduced milk or meat production; or from studies that produce specific data on system components that are then used in simulation models to predict those losses. An example of this is the study by Jonsson (2006) [16], who estimated that; on average, each engorged female tick is responsible for the loss of 1.37 g of body weight in *Bos taurus* cattle. Similarly, the comparable value for *B*. *taurus* × *B*. *indicus* cattle is 1.18 g. Other studies have also observed that in tick-infested animals, each engorged female tick was responsible for a decrease of 8.9 ml of daily milk production and 1.0 g of body weight [17].

Another approach to estimate losses is the economic systems approach, in which all production traits and their interactions are controlled simultaneously. The farming systems approach is useful for quantifying the effects of tick control, as the determinants of tick control effects are extremely complex. Indeed, Ocaido et al. (2009) [18] in Uganda estimated that costs for controlling ticks and TBD constitute between 73.8% and 85.6% of total disease control costs, with tick control alone accounting for between 83.1% and 87.9% of those costs. In this sense, in general, production costs are directly influenced by treatment costs and by farm care on the health status of animals, and such parameters are related to farm profitability.

The present study focused on cattle milk production in subtropical areas of Ecuador. The aim of this study was to estimate the costs of inputs involved in cattle milk production in order to evaluate the influence of acaricide resistance in the components of the production costs on cattle farms. This information will support and allow the development of information campaigns that will help to raise awareness among farmers and target key messages on tick control guidance. It will also allow the creation of tick control programs focused on the reality of small producers in ecologically vulnerable subtropical areas since the main reasons for the failure or lack of sustainability of control programs have been the economic limitations of the farmers [19]. Control program guidelines will help the producer optimise costs, maintaining a low tick infestation level that does not decrease livestock production.

## Materials and methods

### Ethics statement

Due to the nature of the study and the low risk to participants, no formal Ethics Committee approval was required. All animals were treated with care, and the usual farm management of tick collection was followed without mistreatment and ensuring animal welfare. The farmers were properly informed and gave their written consent before starting tick collection from their animals. The survey collected on each farm was coded with numbers and letters according to the farm and area visited.

### Study area and sampling design

This study is part of the project “Socio-eco-epidemiology of ticks, tick-borne parasites, acaricide resistance and residual effects of acaricides in Ecuadorian tropical livestock: environmental, animal and public health impacts”; and focuses on the estimation of economic losses caused by tick infestation. Between November 2020 and March 2021, 139 dairy cattle farms were visited in two sub-tropical areas of Ecuador located in the occidental and oriental foothills in the Ecuadorian Andes, one is in the Quijos River Valley, and the other is in the northwest of Pichincha province. Both places are zones of dairy production and are near to rainforest biodiversity reserves. Due to the absence of a sampling frame, the farms were selected using the snowball sampling technique [20]. However, only 105 farms were considered for this study because they had the necessary information to estimate all the production costs.

### Economic analysis

To analyse the information and identify the revenues and expenses of the cattle farms, the data of an entire year was collected through a previously validated survey using the Epicollect-5 [21] mobile application. The survey was previously validated in the field and by national and international experts. In addition to this, on each farm, the storage place of veterinary drugs was visited. A record of the drugs used, presentation, price, and quantity used for one year was filled out on each farm. The economic data are expressed in US dollars (USD) and correspond to the 2020–2021 fiscal period.

### Expenses

**Fig 1:**
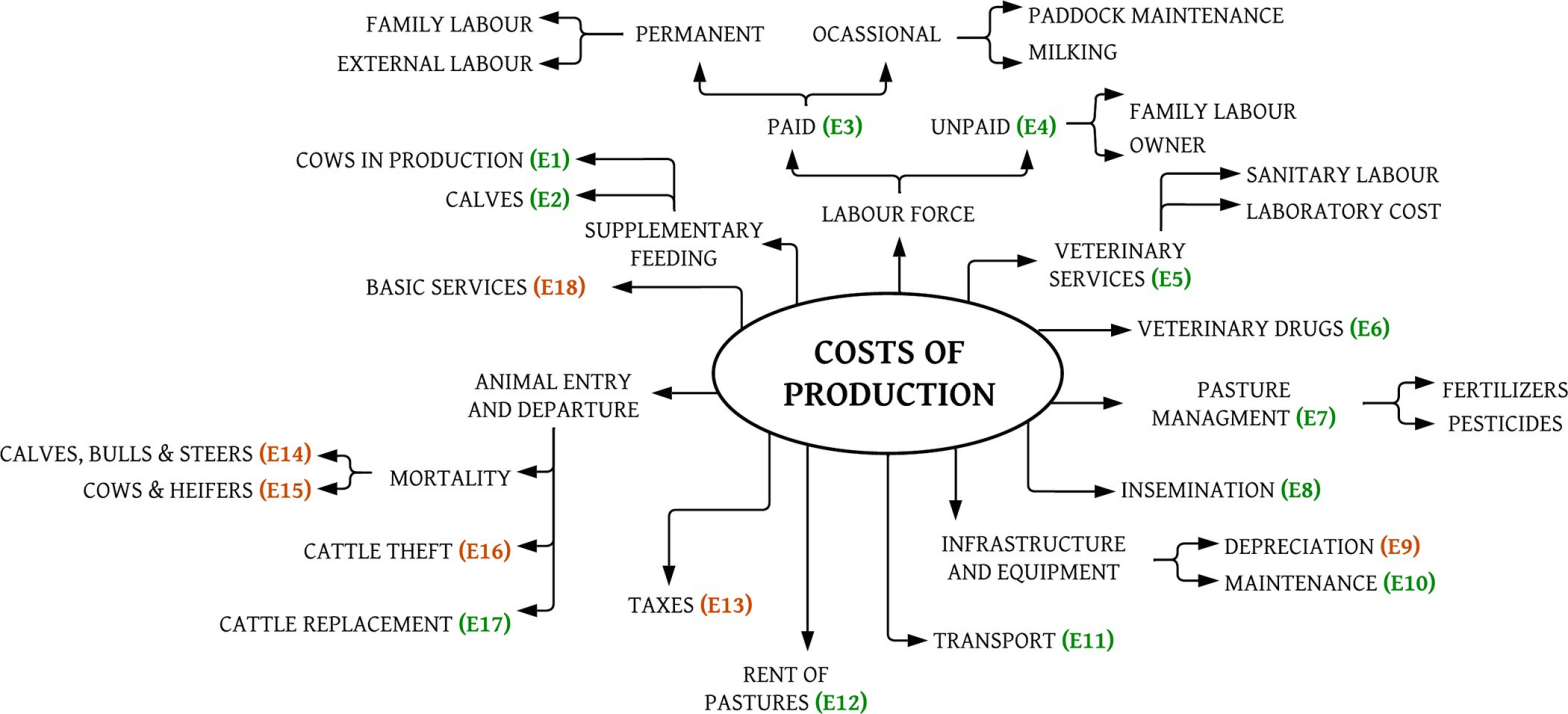
Costs of production associated with milk production in the study area. E1 to E18 are a codification of costs used in the different equations (see afterward). The green coding corresponds to variable costs, and the red coding to fixed costs.

The costs used to determine the total cost of livestock production are described in Fig 1.

Costs were classified as variable (VC) and fixed (FC). In addition to the costs described in Fig 1, information on farming loans was available. However, this information was not taken into account since this cost was included in economic costs E_9_ and E_17_.


$$VC=E_{1}+E_{2}+E_{3}+E_{4}+E_{5}+E_{6}+E_{7}+E_{8}+E_{10}+E_{11}+E_{12}+E_{17}$$ (Eq 1)

$$FC=E_{9}+E_{13}+E_{14}+E_{15}+E_{16}+E_{18}$$ (Eq 2)

The total livestock production cost (TC) corresponds to fixed plus variable costs. Two scenarios were considered, one without the cost of subsidised veterinary services (TC_A_) and one with subsidized veterinary services (TC_B_). It was necessary to consider this division given the fact that local autonomous governments use to support farmers with veterinarians or veterinary technicians. These services are free of cost and are available to farmers who wish to use them. There are several methodologies to calculate the cost of production of a litre of raw milk. However, this study is based on the actual costs directly related to milk production throughout the year (costs of cows and heifers) [22, 23]. For the calculation of the total cost of milk production (TCM), calf feeding costs (E2), mortality (E_14_, E_15_), and cattle theft (E_16_) were excluded (red coded in Eqs 1 and 2), as data on the purchase of replacement animals were used instead. The lack of animals due to mortality is reflected in the decrease in litres of milk delivered and animals sold. Scenario A was used for this calculation.

*Supplementary feeding (E*_*1*_ *and E*_*2*_*)*. Livestock feed includes all costs related to supplementation by concentrate, silage, hay, and by-products (beer bran, molasses); also, mineral salts received by cows in production at milking time. In calves, supplementary feeding includes the cost of concentrate and milk replacer if purchased.

*Labour force (E*_*3*_ *and E*_*4*_*)*. The cost of the labour force includes both paid (E_3_) and unpaid (E_4_) labour. Paid labour can be permanent or occasional. Permanent paid labour included workers who perform daily activities during a specific number of hours and receive a monthly salary, and family members receiving compensation.

Occasionally, the paid labour force performs certain activities such as milking or paddock maintenance. Occasional personnel employed for milking are also called "permanent occasional" since they only come daily for milking. They are paid daily, weekly, biweekly or monthly. The occasional labour force is employed for paddock maintenance operations such as equalisation cuts or paddock cleaning. This service is performed for one week or one month per year, and they are generally paid just for their service, regardless of the time used. Then, based on the number of days and hours of work, the payment for one-day work (8 hours per day) was determined.

In the case of the unpaid owner and family labour, the opportunity cost of a permanent day’s work in the study areas was used. The number of family workers employed was estimated based on the literature. One person is required to milk 18 animals (manual milking) or 25 animals in the case of mechanical milking [24].

*Veterinary services (E*_*5*_*)*. Both real and state-subsidized costs were considered. In the case of state-subsidized veterinarians, the average price of visiting private veterinarians, as reported by respondents, was considered as the opportunity cost assigned.

*Veterinary drugs (inputs) (E*_*6*_*)*. The cost of veterinary supplies includes antibiotics, anti-inflammatories, antiseptics, sanitisers, hormones, vaccines, repellent spray, hemo-parasiticides, vitamins, and minerals (application parenteral), and inputs used in milking (teat sealants, detergents for milking equipment, among others), internal parasiticides and acaricides. The cost for acaricides includes acaricides used parenterally or topically (spray or pour-on).

The price of each drug was collected and consolidated in three different ways. 1. Hard paper survey carried out during fieldwork. 2. Retail price obtained from the catalogue of each distribution company. 3. Interviews at agricultural warehouses in the study zones (8 agricultural warehouses were visited in each zone). The interviews in agricultural warehouses and catalogues consultations were carried out to complete missing prices and corroborate the information obtained in the field. Since the visit to agricultural warehouses took place one year after the field visit, the 2.56% corresponding to annual price inflation in January 2022 was reduced concerning January 2021 [25].

*Pasture management (E*_*7*_*)*. It includes all pesticides, organic fertilisers, and chemicals fertilisers used to maintain the paddocks. The costs of equalisation cut and maintenance of paddock fences were placed in occasional labour. It is usually done in the areas once or twice a year.

*Insemination (E*_*8*_*)*. These are the costs for purchasing straws and filling the nitrogen tank if the farm implements this. This cost is included in veterinary services (E_5_) if a veterinarian or technician performs insemination.

*Infrastructure and equipment (E*_*9*_ *and E*_*10*_*)*. Those values correspond to the cost of depreciation (E_9_) and annual maintenance (E_10_). Infrastructure includes stables, corrals, cattle handling systems, and storage warehouses. The equipment consists of milking equipment, cooling tanks, electric fences, irrigation equipment, fumigation pumps, scythes, grass shredders, biodigesters, and other equipment used for livestock farming. Depreciation was calculated by using a straight-line method, according to the years of the useful life of each asset reported by farmers during the survey. For infrastructure, the average time of useful life was 20 years (infrastructure) and for equipment 11 years.

*Transport (E*_*11*_*)*. These are the costs destined to cover vehicle rental expenses or fuel, as long as they are used in the entry or exit of animal feed, animals, or any input related to livestock raising.

*Rent of pastures (E*_*12*_*)*. This cost was included because it is a common practice in the study areas. On farms where there are not enough paddocks, farmers rent paddocks from neighbouring properties.

*Taxes (E*_*13*_*)*. Corresponds to the payment of the land tax.

*Animal entry and departure (E*_*14*_*-E*_*17*_*)*. The entry and departure of animals from the farm corresponds to the entry of replacement animals, and the departure is due to mortality on the farm and cattle theft.

Information reported by the farmer on sick or dead animals was used to determine the cost of mortality (E_14_ and E_15_). The principal diseases present in the study areas and reported by local veterinarians were used. For each disease, information was available on the number of animals affected, age, and mortality. The cost of sale of the animals corresponds to the average reported in each zone. The mortality costs per farm were limited to 3.00%, corresponding to the average mortality in the study area. Cattle theft (E_16_) was also considered, as it is usually experienced by cattle farmers in the area. The annual depreciation (straight-line) value of the animals was taken into account in the departure of the animals. In the case of cows, the lost remuneration for the litres of milk they stopped producing per year and the cost of a newborn calf was added.

For the calculation of animal replacement (E_17_), the proportion of the cost of the animal about the average useful life of the animal was used. The valuable life corresponds to the average reported in the study areas. Indeed, a life expectancy of 9 years was considered for cows and 5 years for bulls.

*Basic services (E*_*18*_*)*. Corresponds to the amount paid for water and electricity.

#### Revenues

**Fig 2:**
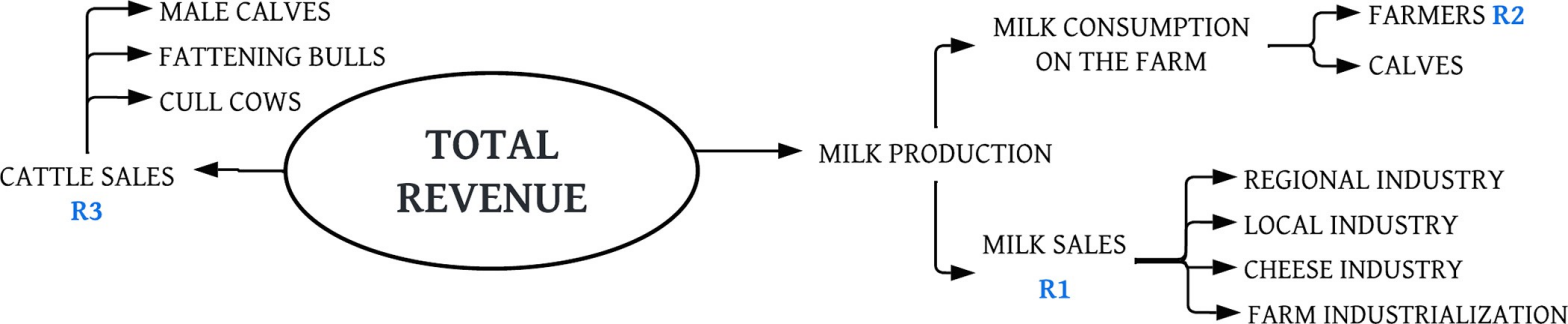
Revenues from a dairy farm in the study area. R1 to R3 are codifications of revenues used in the different equations (see after).

Revenues correspond mainly to milk produced and complementarily to cattle sales (Fig 2).

For the calculation of total revenue (TR) from the sales of milk, the average number of cows in production, the average milk production per day (litre/cow/day), and the price received for the sales of milk were used. We averaged cow/day production in the dry and rainy seasons. Revenue from milk consumed on the farm in the form of raw milk or milk by-products was based on the opportunity cost in the area. The consumption of milk per calf was not used to calculate total revenue because if it is not used; a milk substitute is purchased, an expense that was considered on farms where it is used.

For the calculation of cattle sales, new-born male calves, on-farm born bulls for fattening, or the sale of old cull animals were included. Similarly, the market value for calves and cull cows was determined using the opportunity cost for each zone.


$$TR=R_{1}+R_{2}+R_{3}$$ (Eq 3)

The Annual Profit (P) is the subtraction between the total revenue and total costs of livestock production.


$$P=TR-{TC}_{L}$$ (Eq 4)

The production costs of a litre of milk (CPM) were calculated dividing TCM to the total amount of litres produced per year.

### Acaricide resistance test

On each farm visited, a minimum of 40 engorged female ticks were collected from five randomly selected animals. The collected samples were transported to the laboratory of applied entomology at the Research Institute of Zoonosis (CIZ) in Central University of Ecuador in Quito, where resistance testing was performed on three acaricides: amitraz, ivermectin, and alpha-cypermethrin, using the larval package test. Samples were tested at the farm level and consisted of two replicates per acaricide plus a control group. Approximately 100 larvae were placed in each package. Mortality readings were taken 24 hours after larval seeding. The average of the two replicates was used to determine the percentage of resistance to the acaricide [26]. Concentrations used for the resistance response were: for alpha-cypermethrin 0.02%, for amitraz 0.1% and for ivermectin at 0.1% [15]. Resistance results were classified into four levels: susceptible, low resistance, medium resistance, and high resistance, as described before [15, 27]. The farms were classified as with and without resistance. Farms considered with resistance have medium and high resistance (Table 1).

**Table 1:** Interpretation of acaricide resistance.

Category	Larvae (Survival %)
Susceptible	<10%
Susceptible with Restriction	10–20%
Emerging Resistant	20–50%
Resistant	>50%

Adapted form Rodríguez-Hidalgo et al., 2017; Junte, 2008 [15, 27]

### Level of tick infestation

The level of infestation at the farm level was determined according to the level of infestation in the animals sampled, and they were classified according to the methodology explained by Paucar et al. (2022) [28]. This variable was expressed as farms with or without high level of tick infestation.

### Statistical analysis

#### Farm typology

The k-means [29] algorithm was used to obtain a typology of the farms using the following variables: level of technology, veterinary control, farm size (ha), use of external paddocks, owner’s level of education, owner’s time dedicated to livestock, the destination of milk delivery, milk delivery price (USD), presence of permanent paid labour force, presence of crops for sale, feed supplementation to cattle, and grass cut. The level of technology was determined by the presence of installations, use of artificial insemination, and type of milking [28]. The presence of veterinary control was determined according to the frequency of visits (never, rarely, sometimes, and permanent) regardless of whether it was subsidised or not. Permanent and frequent visits were considered as the presence of veterinary control. The presence of crops on the farm was only considered for sale; crops for self-consumption were not considered. The analysis was performed using R [30]. Elbow Method [31] was used to determine the optimal number of clusters (groups). The packages FactoMineR [32] and Factoextra [33] were used. Both numerical and categorical variables were recategorised into ordinal variables for use in the k-means algorithm.

#### Decision tree analysis

A decision tree was used to determine the impact of management variables on economic variables. Decision trees have been widely used in animal health economic analyses [34–37]. Herd management variables used for the analyses were: technology level, herd size, use of paddocks outside the farm boundaries (external paddocks), the use of manual removal of ticks, the study area, presence of high level of tick infestation (farm level), and presence of tick resistance to three acaricides (Table 2). These variables were selected because of their importance in a previous study [28] or because of their common use in livestock management.

**Table 2:** Variables used to construct decision trees.

Explanatory variables	Categories
Technology level	Non-technified Semi-technified Technified
Herd size	1–20 animals 21–70 animals >71 animals
External paddocks	Yes No
Manual removal of ticks	Yes No
Study area	Quijos river valley (Area 1) Northwest of Pichincha (Area 2)
Presence of high level of tick infestation	Yes No
Amitraz resistance	Yes No
Ivermectin resistance	Yes No
Alpha-cypermethrin resistance	Yes No

The economic variables for model 1, 3 and 4 are expressed in %. Model 1 used the cost of acaricide treatment versus the total production cost. Model 3 used the cost of drugs used for tick control against the cost of drugs and other inputs (E_6_), and for model 4 used the relationship between the cost of drugs used to control ticks and the labour force used in acaricide treatments versus the sanitary costs. For model 2, the economic variable is expressed in American Dollars (USD) of acaricide treatment per animal.

The cost of acaricides, hemo-parasiticides, and repellents was used to calculate the cost of drugs used for the presence of ticks. The labour force cost was determined by the time taken to apply the acaricide treatments and the labour force cost in each study area. The sanitary cost includes the cost of drugs and inputs, veterinary services, and the cost of the labour force used for sanitary work (vaccination, deworming, bathing, castration, identification, dehorning, and insemination). To calculate the annual cost per animal, the total costs of acaricide treatment was divided by the number of adult cattle (female or male animals over 1 year old) present on each farm.

In addition, a decision tree was constructed between the presence of high level of tick infestation (response variable), resistance to three acaricides, and level of technology, as these are essential variables to understand this relationship.

Decision trees were built using the rpart and rpart.plot packages [38, 39] packages in R. Validation was performed using a split of the database for training the model (75% of random data) and the rest for testing the model, which is a procedure that reveals the performance of the model with new data [40]. For model prediction, we ran the decision trees procedure several times (one hundred times) and chose only the models in which the prediction response and observation were in concordance. It means models in which the slope of the line formed between observed vs. prediction response was close enough to one and the intercept close enough to zero, and where the differences between prediction and observation did not differ significantly according to Bland-Altmant agreement between two quantitative measurements (package BlandAltmanLeh) [41]. We also chose the models also with minimum mean square errors.

## Results

### Tick infestation and acaricide resistance

Of the 105 farms analysed, 45.71% (48 farms) were had high tick infestation. In general, in farms with and without high level of infestation, the resistance to alpha-cypermethrin was about 53.33%, and it was the highest level of resistance compared to the other acaricides (Table 3). The levels for amitraz and ivermectin resistances were 49% and 37%, respectively. Table 4 shows the results of combined resistances. Thirty-two percent of the farms with or without high level of tick infestation are resistant to both amitraz and alpha-cypermethrin. There was no significant difference between the presence of high level of infestation and resistance in single and combined resistances.

**Table 3:** Contingency table between the high level of infestation and resistance to acaricides.

High level of tick infestation	AM resistance		IV resistance		CY resistance	
	*p-*value = 0.85		*p-*value = 1.00		*p-*value = 0.33	
	No	Yes	No	Yes	No	Yes
**No** (N = 57)	26.67% (N = 28)	27.62% (N = 29)	34.29% (N = 36)	20.00% (N = 21)	22.86% (N = 24)	31.43% (N = 33)
**Yes** (N = 48)	23.81% (N = 25)	21.90% (N = 23)	28.57% (N = 30)	17.14% (N = 18)	23.81% (N = 25)	21.90% (N = 23)
**Total**	50.48%	49.52%	62.86%	37.14%	46.67%	53.33%

AM = Amitraz; IV = Ivermectin; CY = Alpha-cypermethrin; N = number of farms; *p*-value was obtained using Fisher’s exact test.

**Table 4:** Contingency table with combined resistance to acaricides.

High level of tick infestation	AM & CY		AM & IV		CY & IV		AM -CY & IV	
	resistance		resistance		resistance		resistance	
	*p-*value = 0.54		*p-*value = 1.00		*p-*value = 0.82		*p-*value = 1.00	
	No	Yes	No	Yes	No	Yes	No	Yes
**No** (N = 57)	35.24% (N = 37)	19.05% (N = 20)	41.90% (N = 44)	12.38% (N = 13)	40.00% (N = 42)	14.29% (N = 15)	42.86% (N = 45)	11.43% (N = 12)
**Yes** (N = 48)	32.38% (N = 34)	13.33% (N = 14)	35.24% (N = 37)	10.48% (N = 11)	35.24% (N = 37)	10.48% (N = 11)	39.05% (N = 41)	6.67% (N = 7)
**TOTAL**	67.62%	32.38%	77.14%	22.86%	75.24%	24.77%	81.90%	18.90%

AM & CY = Farms with resistance to amitraz and alpha-cypermethrin; AM & IV = Farms with resistance to amitraz and ivermectin; CY & IV = Farms with resistance to alpha-cypermethrin and ivermectin; AM, CY & IV = Farms with resistance to amitraz, ivermectin, and alpha-cypermethrin; *p*-value was obtained using Fisher’s exact test.

**Fig 3:**
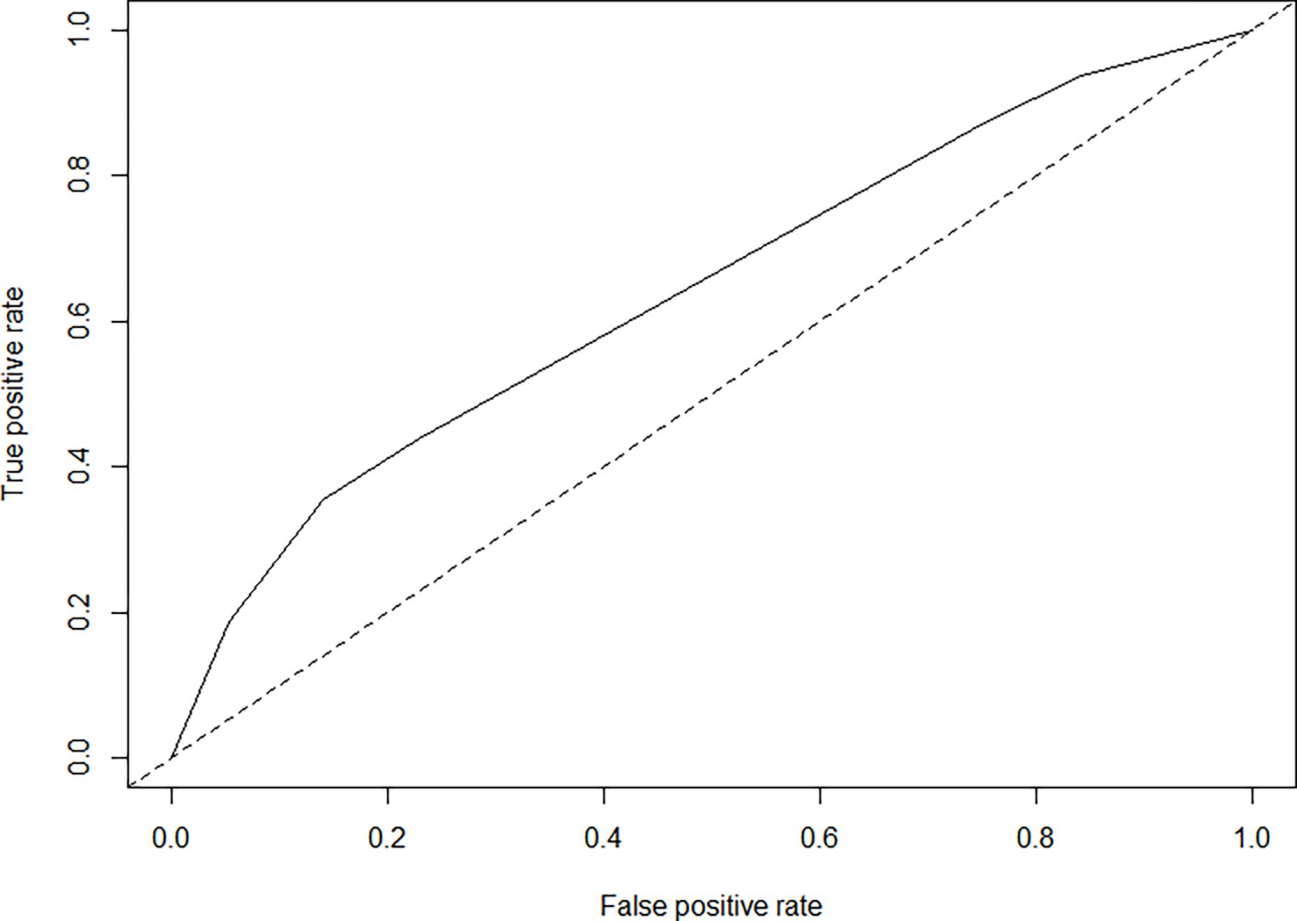
Receiver operating characteristic (ROC) curve of acaricide resistance, presence of high level of tick infestation, and level of technology.

**Fig 4:**
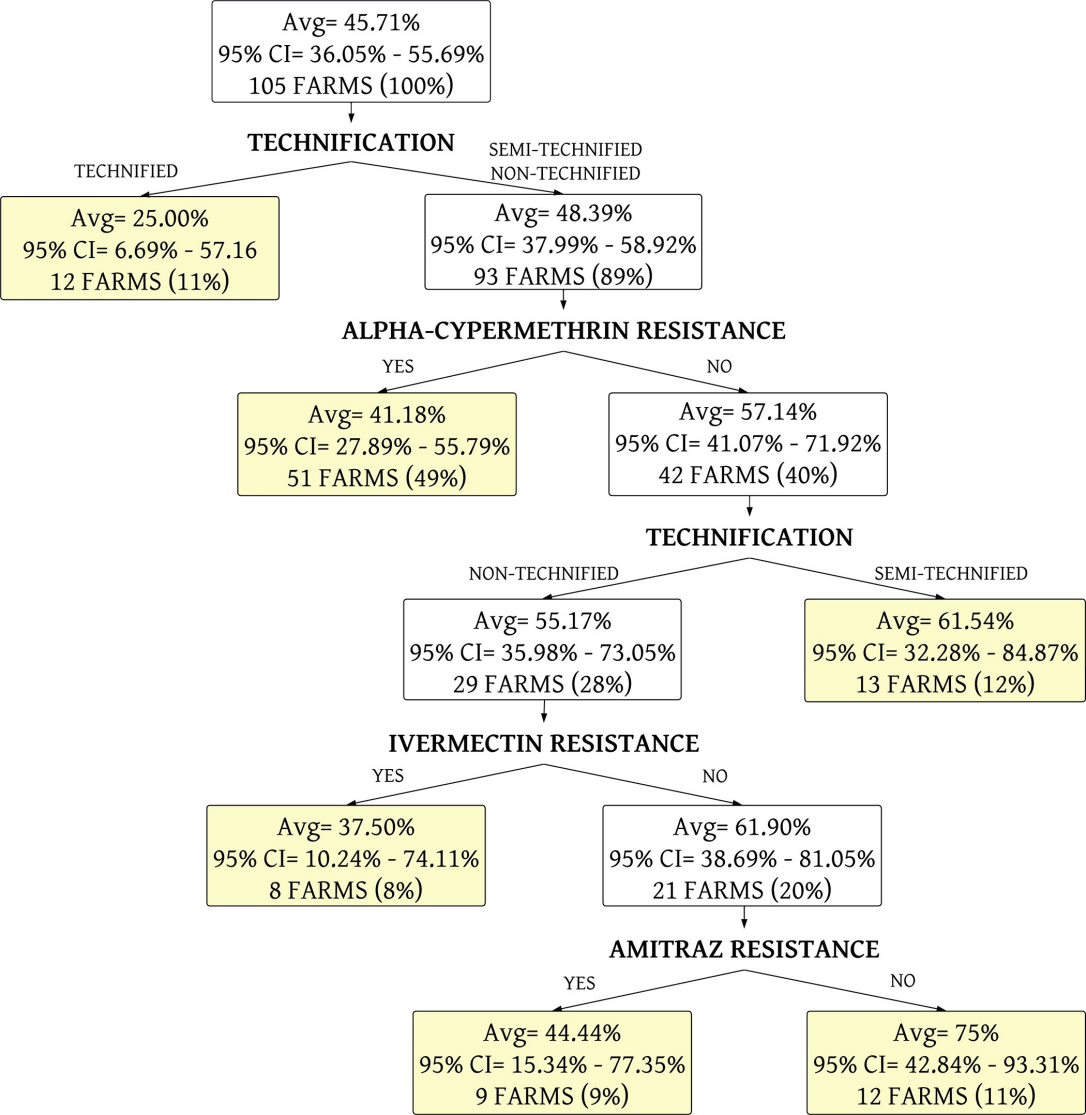
Decision tree for high level of tick infestation on animals using the levels of acaricide resistance, and level of farm technology. The yellow boxes represent the terminal nodes of the decision tree. The average (Avg) corresponds to the % of infested farms. CI is the confidence interval.

A decision tree was constructed for the presence of high level of tick infestation in relation to the presence of tick resistance to three acaricides, and also with the level of technology. It presented an area under the receiver operator characteristic curve (AUC-ROC) of 0.64 (95% CI: 0.54–0.74) (Fig 3), showing that those results are not random. In this model (Fig 4), it was observed that when a farm is sensitive (farm without resistance) to the acaricides, there were higher levels of tick infestation in the farm, but low level of technology.

### Farm typology

The five groups or types of farmers were formed using means of the variables described in Table 5. Group 1 has 21 of 105 farms (20.00%), group 2 has 17 of 105 farms (16.19%), group 3 has 27 of 105 farms (25.71%), group 4 has 23 of 105 farms (21.90%), and group 5 has 17 of 105 farms (16.19%). The groups were divided into small, medium, and large farms according to farm size. The medium farms were classified into three groups according to their level of technology and whether they cultivate and sell crops. Concentrate supplementation to cattle is done in four out of five groups, in contrast to supplementation with cutting pastures, which is only done in the group of large farms. Small farms delivered milk to the local industry, medium farms to the cheese industry, and large farms to the milk regional industry. Milk prices were similar in four out of five groups (local industry and cheese factories). However, the large farms obtained better remuneration per litre of raw milk due to their main delivery destination is the regional industries.

**Table 5:** Farm typology in the study area.

Variable	Group of farm				
	1	2	3	4	5
Farm size	1-20ha	21-45ha	21-45ha	21-45ha	>46ha
External paddocks	Yes	Yes	No	No	No
Level of technification	Non- technified	Non- technified	Non- technified	Semi- technified	Technified
Veterinary control	Yes	No	No	Yes	Yes
Permanent paid labour force	No	No	Yes	No	Yes
Crops for sale	No	Yes	No	No	No
Concentrate supplementation	Yes	No	Yes	Yes	Yes
Grass cut	No	No	No	No	Yes
Milk delivery price (USD)	0.37	0.37	0.38	0.37	0.47
Destination of milk delivery	Local industry	Cheese industry	Cheese industry	Cheese industry	Regional industry
Owner’s level of education	Primary school	Primary school	High school	High school	University
Owner’s time dedicated to livestock	3.00 hrs.	4.38 hrs.	1.75 hrs.	5.00 hrs.	1.16 hrs.

Primary school, including farmers without formal education; High school, including farmers with unfinished university education.

The five groups are described below.

#### Group 1—Small, non-technified cattle farms

Farms in this group have an area of 1-20ha, requiring the rental of external paddocks to feed their cattle, especially for dry cows. Their level of technology is low, but they have frequent veterinary controls. Since they are small farms, do not require permanent labour, but 95.23% of these farms use occasional personnel for specific tasks such as milking or pasture maintenance. In addition, 80.95% of these farms use unpaid family labour. The owner has an elementary school education and is involved in animal management, spending an average of 3 hours per day on it. Almost equal numbers of men (52.38%) and women (47.52%) are managers of livestock.

#### Group 2—Medium, non-technified cattle and agricultural farms

Farms in this group have an area of 21-45ha, requiring the rental of external paddocks to feed their cattle. The animals do not receive supplementary feed. Their level of technology is low, and they do not have veterinary control. They do not have permanent labour. However, 64.70% of these farms use occasional labour for pasture maintenance. In addition, 88.24% of these farms use unpaid family labour. The owner has a high school education and is involved in animal management. These farms, apart from being livestock farms, are dedicated to the cultivation and sale of palm hearts, bananas, coffee, and cocoa, among others. The farmer his time spending, an average of 4.38 hours per day. Almost equal numbers of men (52.94%) and women (47.06%) are managers of livestock.

#### Group 3—Medium, non-technified cattle farms

Farms in this group have an area of 21-45ha and have the necessary pastures to feed their cattle. Their level of technology is low, and they do not use veterinary control. They have a permanent labour force; in addition, 70.37% of the farms have an occasional labour force for pasture maintenance in general. Unpaid family labour is only used on 22.22% of farms. The owner has a high school education and generally participates only in milking activities, dedicating an average of 1.75 hours per day to the herd. The managers of the livestock on most of the farms are men (62.96%).

#### Group 4—Medium, semi-technified cattle farms

Farms in this group have an area of 21-45ha and have the necessary paddocks to feed their cattle. Their level of technology is medium, and they use veterinary control. They do not have a permanent labour force; however, 73.91% of these farms use occasional personnel for pasture maintenance. In addition, 82.61% of these farms use unpaid family labour. The owner has a high school education and is involved in animal management, dedicating an average of 5 hours per day to these tasks. The managers of the livestock on most of the farms are men (78.26%).

#### Group 5—Large, technified cattle farms

Farms in this group have an area of over 46ha and the necessary paddocks to feed their cattle. They are technified, have veterinary control, and the animals receive concentrated feed and cut pasture, and mineralised salt. They have a permanent labour force, and, in addition, 76.47% of the farms have occasional labour for pasture maintenance in general. Unpaid family labour is used on 29.41% of farms. The owner has a university education and is not involved in animal management; they spend an average of 1.16 hours per day on these administrative activities. The managers of the livestock on most of the farms are men (70.59%).

### Economic analysis

The main expenses in livestock farming are labour and supplementary livestock feed (Table 6). In the groups of small and medium farms (groups 1 to 4), the main costs correspond to the labour force, while in large farms (group 5), it is supplementary feeding. In general, within labour, 54.18% of the expenses correspond to paid labour and 45.82% to unpaid family labour (those activities were set as part of the opportunity costs). However, when analysed by groups, the cost of paid labour in groups 5 and 3 corresponds to 89.56% and 80.17%, respectively. In contrast, groups 1 (74.02%), 2 (88.54%), and 4 (92.91%) correspond to unpaid labour, either from the owner or the family.

**Table 6:** Fixed and variable costs in livestock farming.

Group		E_1_-E_2_	E_3_-E_4_	E_5_(A)	E_5_(B)	E_6_	E_7_	E_8_	E_9_,E_10_	E_11_	E_12_	E_13_	E_14_-E_17_	E_18_
**1**	AVG	2205.11	4754.72	47.80	153.60	706.07	54.55	32.38	409.71	410.00	293.33	36.24	494.10	94.29
	SD	1696.56	2528.09	51.14	159.66	693.86	141.85	72.17	404.60	731.17	474.22	42.62	532.55	139.18
	%A	23.12	49.85	0.50	-	7.40	0.57	0.34	4.30	4.30	3.08	0.38	5.18	0.99
	%B	22.86	49.30	-	1.59	7.32	0.57	0.34	4.25	4.25	3.04	0.38	5.12	0.98
**2**	AVG	756.38	4186.77	36.35	104.45	645.24	7.97	36.47	330.69	599.65	156.47	96.35	557.51	289.41
	SD	449.89	865.90	67.61	168.40	431.34	20.99	119.21	349.26	1285.45	307.75	104.69	992.15	372.72
	%A	9.82	54.38	0.47	-	8.38	0.10	0.47	4.30	7.79	2.03	1.25	7.24	3.76
	%B	9.74	53.90	-	1.34	8.31	0.10	0.47	4.26	7.72	2.01	1.24	7.18	3.73
**3**	AVG	2126.13	6854.49	147.68	227.92	1147.76	54.95	33.70	613.11	460.74	50.00	113.67	750.23	241.78
	SD	1475.81	3907.01	290.61	325.31	929.57	148.40	132.46	437.34	277.32	196.61	140.16	752.33	238.07
	%A	16.88	54.43	1.17	-	9.11	0.44	0.27	4.87	3.66	0.40	0.90	5.96	1.92
	%B	16.77	54.08	-	1.80	9.06	0.43	0.27	4.84	3.64	0.39	0.90	5.92	1.91
**4**	AVG	1682.27	4732.99	54.30	269.87	621.69	42.05	19.78	406.54	555.30	26.09	73.00	600.53	122.61
	SD	1613.55	1189.27	90.66	856.30	372.70	138.07	67.09	377.06	731.79	125.11	67.79	906.67	137.11
	%A	18.82	52.96	0.61	-	6.96	0.47	0.22	4.55	6.21	0.29	0.82	6.72	1.37
	%B	18.38	51.71	-	2.95	6.79	0.46	0.22	4.44	6.07	0.29	0.80	6.56	1.34
**5**	AVG	13409.98	10261.82	683.12	716.16	2787.32	94.97	506.21	3579.03	963.82	0.00	366.24	1130.27	1256.00
	SD	1613.55	1189.27	90.66	856.30	372.70	138.07	67.09	377.06	731.79	125.11	67.79	906.67	137.11
	%A	38.27	29.29	1.95	-	7.95	0.27	1.44	10.21	2.75	0.00	1.05	3.23	3.58
	%B	38.24	29.26	-	2.04	7.95	0.27	1.44	10.20	2.75	0.00	1.04	3.22	3.58
**Total**	AVG	3649.84	6089.57	175.92	281.30	1128.28	50.92	107.34	961.65	575.25	102.57	127.36	696.54	358.10
	SD	10998.55	5585.50	950.68	934.58	2464.00	258.29	612.57	1940.44	903.72	0.00	506.22	1579.82	1220.47
	%A	26.03	43.42	1.25	-	8.05	0.36	0.77	6.86	4.10	0.73	0.91	4.97	2.55
	%B	25.83	43.10	-	1.99	7.99	0.36	0.76	6.81	4.07	0.73	0.90	4.93	2.53

%A = Percentage that corresponds to each expenditure, with respect to the total cost of livestock production, within Scenario A (cost of veterinary services without state subsidies); %B = Percentage that corresponds to each expenditure, with respect to the total cost of livestock production, within Scenario B (cost of veterinary services with state subsidies); AVG, average; SD, standard deviation.

Veterinary services were evaluated in two scenarios, without (Scenario A) and with subsidy (Scenario B). The subsidised veterinary technician is in charge of insemination, dehorning, castration, oestrus synchronisation, diagnosis, and treatment of diseases, although they are not always licensed professionals. In addition, to perform these activities, the private veterinarian focused on reproductive check-ups (gynaecological-obstetrical). The cost of veterinary services in the overall analysis ranged from 1.25% (Scenario A) to 1.99% (Scenario B) of the total cost of livestock production. Nevertheless, when analysed by groups, it represents an economic advantage for groups 1, 2, and 4. In groups 3 and 5, costs with or without veterinary services varied very little.

The average annual profit varies little between the two scenarios. The average production price per litre of milk is 0.30 USD. The cost of production is highest in the small farms (0.34 USD) and lowest in the medium, non-technified cattle farms (0.25 USD).

In general, the cost of milk production is 0.30 USD (95% CI: 0.17–0.43). In farms from groups 1 and 2, the production cost per litre of milk in higher, in contrast to farms in groups 3, 4, and 5 (Table 7). However, the difference was only significant between groups 1 and 3 (*p-*value = 0.03).

**Table 7:** Economic evaluation according to farm typology.

		Group 1	Group 2	Group 3	Group 4	Group 5	Total
**TC**_**A**_	AVG	9538.31	7699.26	12594.24	8937.16	35038.78	14023.34
	SD	5279.02	2706.80	5529.43	3117.79	3117.79	21272.13
**TC**_**B**_	AVG	9644.11	7767.36	12674.48	9152.73	35071.82	14128.73
	SD	5287.09	2669.62	5656.09	3216.60	3216.60	21255.63
**AMP**	AVG	31641.61	26908.78	51767.24	37490.71	120349.55	51693.97
	SD	21392.84	15004.43	23953.17	30143.71	71379.06	47316.52
**TR**	AVG	13704.66	11950.53	21476.78	16107.37	64103.67	24105.35
	SD	9429.39	7067.14	8905.42	14570.78	48902.19	27944.26
**P**_**A**_	AVG	4166.35	4251.26	8882.54	7170.21	29064.89	10082.01
	SD	4166.35	4251.26	8882.54	7170.21	29064.89	10082.01
**P**_**B**_	AVG	4060.55	4183.16	8802.30	6954.64	29031.85	9976.63
	SD	4980.53	6645.17	7805.33	13023.12	32107.77	17191.80
**CPM**	AVG	0.34	0.32	0.25	0.30	0.30	0.30
	SD	0.15	0.14	0.09	0.14	0.11	0.13

TC_A_ = Total livestock production cost in Scenario A; TC_B_ = Total livestock production cost in Scenario B; AMP = Annual Milk Production; TR = Total revenue; P_A_ = Annual Profit in Scenario A; P_B_ = Annual Profit in Scenario B; CPM = Cost of production of a litre of milk; AVG, average; SD, standard deviation.

The survey also revealed the repercussions suffered by farmers due to the COVID-19 pandemic. A total of 69.52% (73 farms) of the farmers stated that their income from milk sales decreased due to the limitations caused by the quarantine in the country, which was more evident at the beginning of it (March 2020 to August 2020). During this period, some milk collection companies did not pay on time or did not collect milk every day. Hence, several farms looked for another company to deliver milk or implement homemade cheese production to sell it. The decrease in the payment per litre of milk and the limitation of the volume of milk received by the milk collection companies was also still occurring at the time of the data collection for this study (Table 8). All these consequences are even more evident in small (Group 1) and medium farms (Group 2, 3, and 4).

**Table 8:** Farms that experienced economic repercussions due to the COVID-19 pandemic.

Effect	Group 1		Group 2		Group 3		Group 4		Group 5	
	Farms	%	Farms	%	Farms	%	Farms	%	Farms	%
Decrease in the price per litre of milk	13	86.67%	12	92.31%	19	90.48%	18	100.00%	3	50.00%
Limited the volume of milk to be commercialised	0	0.00%	0	0.00%	0	0.00%	1	5.56%	2	33.33%
The company did not collect the milk	6	40.00%	3	23.08%	5	23.81%	5	27.78%	1	16.67%
Changed milk collection company	3	20.00%	1	7.69%	0	0.00%	2	11.11%	0	0.00%
Made cheeses for marketing	3	20.00%	1	7.69%	4	19.05%	3	16.67%	0	0.00%
Milk sales payments not on time	0	0.00%	0	0.00%	1	4.76%	0	0.00%	1	16.67%
Sold cattle	2	13.33%	1	7.69%	3	14.29%	1	5.56%	1	16.67%
Dry Cow Therapy	2	13.33%	1	7.69%	0	0.00%	1	5.56%	0	0.00%

Group 1 = 71.43% (15 of 21 farms); Group 2 = 76.47% (13 of 17 farms); Group 3 = 77.78% (21 of 27 farms; Group 4 = 78.26% (18 of 23 farms); Group 5 = 35.29% (6 of 17 farms).

#### Impact of acaricide resistance and tick infestation on total costs

**Fig 5:**
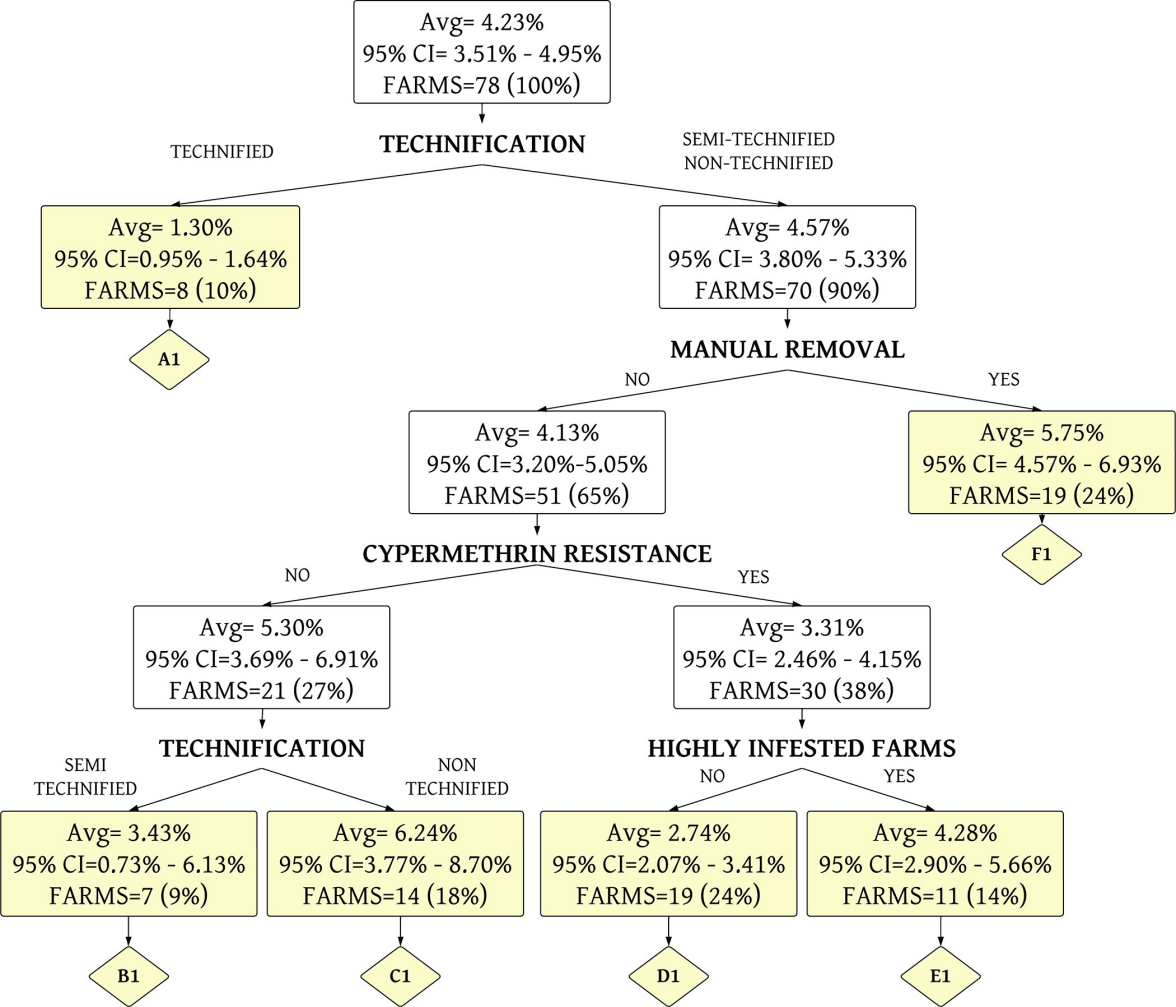
Decision tree analysis of the percentage of total costs allocated to acaricide treatment (Model 1). A1 until F1 are the terminal nodes of the tree (yellow boxes). The average (Avg) corresponds to the % of total costs destined to the acaricide treatment. CI is the confidence interval.

The percentage of acaricide treatment costs to management variables was grouped into six homogeneous terminal nodes using a decision tree analysis (Fig 5 and S1 Table). This model presented a determination coefficient (R^2^) of 0.26 and a mean squared error (MSE) of 2.74% in training data. The R^2^ was 0.21, and MSE was 3.70% in testing data.

The six terminal nodes are described below.

*Terminal node A1*. This group corresponds to technified farms (100.00%), with veterinary control in all farms; with high level of tick infestation in 25.00% of farms. The main problem is resistance to amitraz in 50.00% of the farms, followed by alpha-cypermethrin (37.50%) and ivermectin (12.50%). Regarding combined resistance, 37.50% of the farms showed resistance to both amitraz and alpha-cypermethrin. Their cost of acaricide treatment is 1.30% of the total annual cost (Average = 30031.33 USD). Most of the farms in this terminal node belong to Group 5.

*Terminal node B1*. This group corresponds to semi-technified farms (100.00%), with veterinary control in 85.71% of cases; with high level of tick infestation in 71.43% of farms. These farms are sensitive to alpha-cypermethrin; its main problem is resistance to ivermectin (42.86%) and amitraz (14.29%). Regarding combined resistance, 14.29% of the farms showed resistance to both amitraz and ivermectin. Their cost of acaricide treatment is 3.43% of the total annual cost (Average = 14804.77 USD).

*Terminal node C1*. This group corresponds to non-technified farms (100.00%), with veterinary control in 57.14% of cases; and with high level of tick infestation in 50.00% of farms. These farms are sensitive to alpha-cypermethrin, and its main problem is resistance to amitraz (42.86%) and ivermectin (35.71%). Regarding combined resistance, 14.29% of the farms showed resistance to both amitraz and ivermectin. Their cost of acaricide treatment is 6.24% of the total annual total cost (Average = 9646.38 USD). Most of the farms in this terminal node belong to Group 3.

*Terminal node D1*. This group corresponds to non-technified farms (68.42%), with veterinary control in 47.37% of cases; and without high levels of tick infestation on the farms. The main problem is resistance to alpha-cypermethrin in all farms, followed by amitraz (57.89%) and ivermectin (47.37%). Regarding combined resistance, 57.89% of the farms showed resistance to both amitraz and alpha-cypermethrin. Their cost of acaricide treatment is 2.74% of the total annual costs (Average = 12004.77 USD). Most of the farms in this terminal node belong to Groups 3 and 4.

*Terminal node E1*. This group corresponds to non-technified farms (72.73%), with veterinary control in 54.55% of cases; and high level of tick infestation in all farms. The main problem is resistance to alpha-cypermethrin in all farms, followed by amitraz (54.55%) and ivermectin (45.45%). Regarding combined resistance, 54.55% of the farms showed resistance to both amitraz and alpha-cypermethrin. Their cost of acaricide treatment is 4.28% of the total annual costs (Average = 9566.59 USD). Most of the farms in this terminal node belong to Group 1 and 2.

*Terminal node F1*. This group corresponds to non-technified farms (68.42%), with veterinary control in 84.21% of cases; and high level of tick infestation in 42.11% of farms. The main problem is resistance to amitraz in 52.63% of the farms, followed by alpha-cypermethrin (42.11%) and ivermectin (31.58%). Regarding combined resistance, 26.32% of the farms showed resistance to both amitraz and alpha-cypermethrin. Their cost of acaricide treatment is 5.75% of the total annual costs (Average = 11499.42 USD). Most of the farms in this terminal node belong to Group 1.

#### Annual cost of acaricide treatment per animal

**Fig 6:**
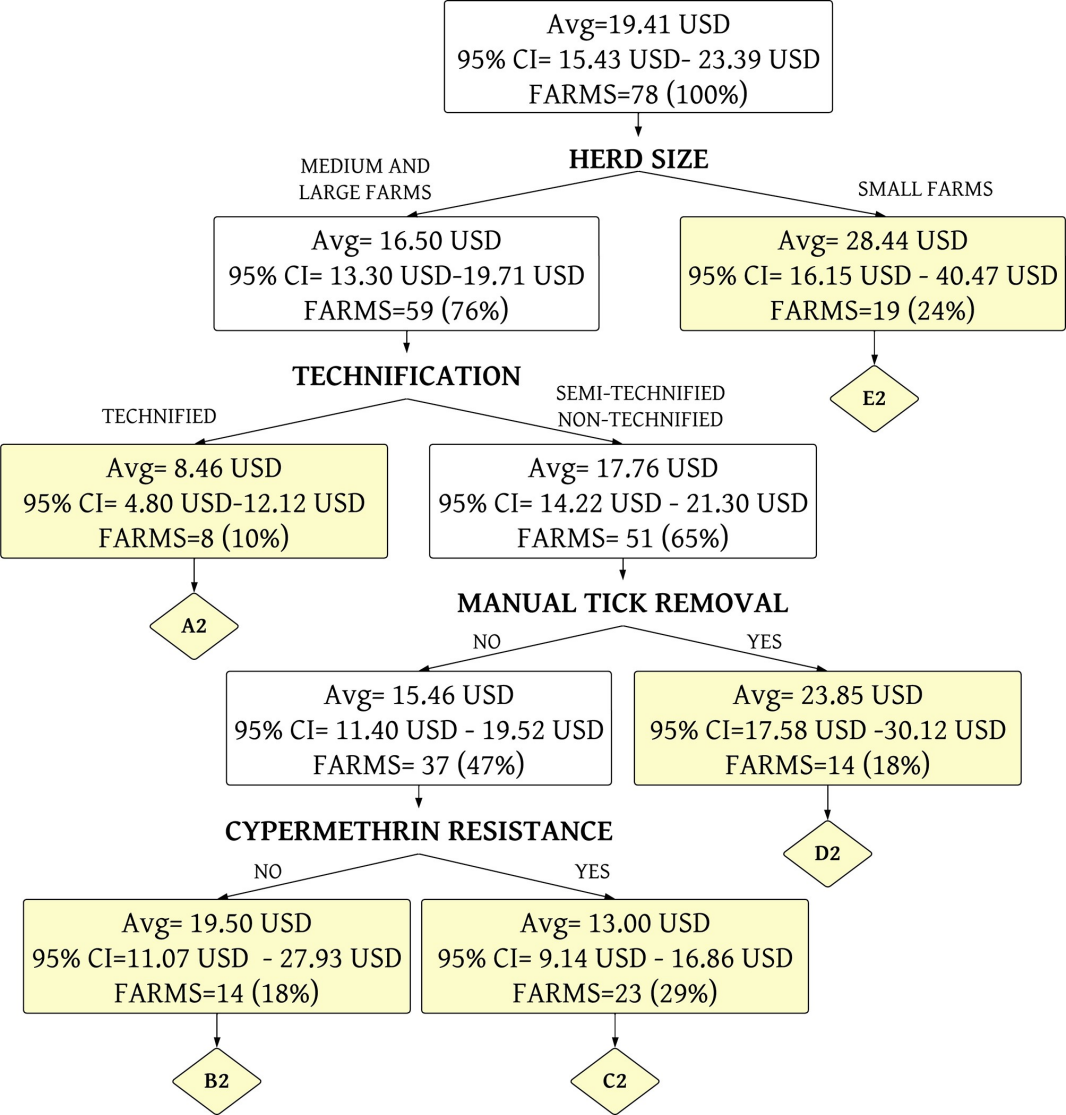
Decision tree analysis of the annual cost of acaricide treatment per animal (Model 2). A2 until E2 are the terminal nodes of the tree (yellow boxes). The average (Avg) corresponds to the annual cost (USD) of acaricide treatment per animal. CI is the confidence interval.

Annual cost in USD of acaricide treatment per animal to management variables was grouped into five homogeneous terminal nodes using a decision tree analysis (Fig 6 and S2 Table). This model presented a R^2^ of 0.16, a MSE of 16.23 USD in training data. The R^2^ was 0.35 and MSE of 10.08 USD in testing data.

The five terminal nodes are described below.

*Terminal node A2*. This group corresponds to technified farms (100.00%), with high level of tick infestation in 37.50% of farms. The main problem is resistance to amitraz in 75.00% of the farms, followed by alpha-cypermethrin (62.50%) and ivermectin (25.00%). The 50.00% of these farms perform manual removal of ticks during milking activities. Pour-on treatments are used on 50.00% of the farms. The 87.50% of the farms use acaricides correctly. Farmers refrain from mixing commercial presentations, overdose or under-dose. On average, acaricide treatment is given every 53.42 days. Their cost of acaricide treatment per animal is 8.46 USD. Most of the farms in this terminal node belong to Group 5.

*Terminal node B2*. This group corresponds to non-technified farms (92.86%), with high level of tick infestation in 57.14% of farms. These farms are sensitive to alpha-cypermethrin; its main problem is resistance to amitraz and ivermectin, with 35.71% in both cases. These farms do not manually remove ticks during milking activities. Pour-on treatments are used on 64.29% of the farms. The 71.43% of farms use acaricides incorrectly. Farmers are mixing commercial presentations (35.71%), overdosing (50.00%) or underdosing (14.29%) acaricides. On average, acaricide treatment is given every 28.52 days. Their cost of acaricide treatment per animal is 13.00 USD. Most of the farms in this terminal node belong to Group 3.

*Terminal node C2*. This group corresponds to non-technified farms (60.87%), with high level of tick infestation in 52.17% of farms. All farms are resistant to alpha-cypermethrin, followed by amitraz and ivermectin, with 47.83% in both cases. These farms do not manually remove ticks during milking activities. Pour-on treatments are used on 26.09% of the farms. The 65.22% of farms use acaricides incorrectly. Farmers are mixing commercial presentations (17.35%), overdosing (56.52%) or underdosing (8.70%) acaricides. On average, acaricide treatment is given every 26.74 days. Their cost of acaricide treatment per animal is 19.50 USD. Most of the farms in this terminal node are medium cattle farms (Groups 2, 3 and 4).

*Terminal node D2*. This group corresponds to non-technified farms (78.57%), with high level of tick infestation in 35.71% of farms. The main problem is resistance to alpha-cypermethrin in 57.14% of the farms, followed by amitraz (50.00%) and ivermectin (42.86%). All farms perform manual removal of ticks during milking activities. Pour-on treatments are used on 35.71% of the farms. The 42.86% of farms use acaricides incorrectly. Farmers are mixing commercial presentations (21.43%), overdosing (35.71%) or underdosing (7.40%) acaricides. On average, acaricide treatment is given every 15.79 days. Their cost of acaricide treatment per animal is 23.85 USD. Most of the farms in this terminal node belong to Group 1.

*Terminal node E2*. This group corresponds to non-technified farms (57.89%), with high level of tick infestation in 52.63% of farms. The main problem is resistance to amitraz in 57.89% of the farms, followed by alpha-cypermethrin and ivermectin with 47.37% in both cases. The 36.84% of these farms perform manual removal of ticks during milking activities. Pour-on treatments are used on 26.32% of the farms. The 42.11% of farms use acaricides incorrectly. Farmers are mixing commercial presentations (42.11%), overdosing (36.84%) or underdosing (5.26%) acaricides. On average, acaricide treatment is given every 19.63 days. Their cost of acaricide treatment per animal is 28.44 USD. Most of the farms in this terminal node belong to Group 1.

#### Impact of acaricide resistance and tick infestation on drug costs (E6)

**Fig 7:**
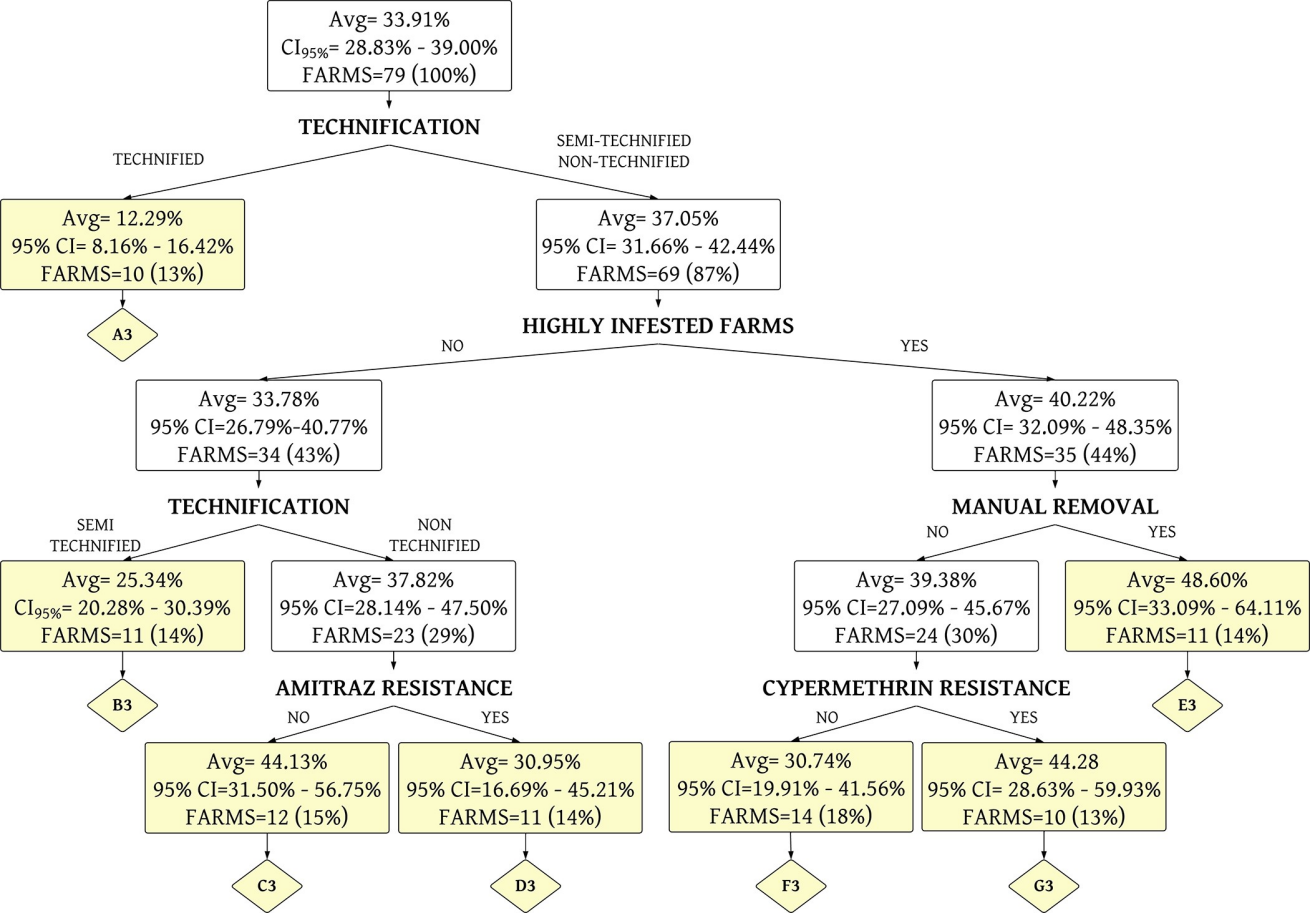
Decision tree analysis of the percentage of drug costs (E6) allocated to tick and TBD control. A3 until G3 are the terminal nodes of the tree (yellow boxes). The average (Avg) corresponds to the % of E6 used for tick and tick-borne diseases control. CI is the confidence interval.

The percentage of acaricide drug costs to management variables was grouped into seven homogeneous terminal nodes using a decision tree analysis (Fig 7 and S3 Table). This model presented a R^2^ of 0.26 and a MSE of 19.54% in training data. The R^2^ was 0.20 and MSE was 20.42% in testing data.

The seven terminal nodes are described below.

*Terminal node A3*. This group corresponds to technified farms (100%), with veterinary control in 90.00% of cases; high level of tick infestation in 20.00% of farms. The main problem is resistance to amitraz in 70.00% of the farms, followed by alpha-cypermethrin (50.00%) and ivermectin (20.00%). Regarding combined resistance, 50.00% of the farms showed resistance to both amitraz and alpha-cypermethrin. Their cost for acaricide drugs is 12.29% with respect to the costs of all veterinary inputs (this annual average cost is 1628.19 USD). Most of the farms in this terminal node belong to Group 5 (S1 Table).

*Terminal node B3*. This group corresponds to semi-technified farms (100.00%), with veterinary control in 72.73% of cases, and they are not highly infested by ticks. Its main problem is resistance to alpha-cypermethrin in 63.64% of the farms, followed by amitraz and ivermectin, with 36.36% in both cases. Regarding combined resistance, 36.36% of the farms showed resistance to amitraz and alpha-cypermethrin. Their cost for acaricide drugs is 25.34% with respect to the costs of all veterinary inputs with an average 969.86 USD per year. The farms belonging to this node are part of small (Group 1) and medium cattle farms (Groups 3 and 4).

*Terminal node C3*. This group corresponds to non-technified farms (100.00%), with veterinary control in 41.67% of cases and are not highly infested by ticks. These farms are sensitive to amitraz. Their main problem is resistance to alpha-cypermethrin in 66.67%, followed by ivermectin with 50.00%. Regarding combined resistance, 25.00% of the farms showed resistance to both alpha-cypermethrin and ivermectin. Their cost for acaricide drugs is 44.13% compared to the costs of all veterinary inputs with average 691.19 USD per year. Most of the farms in this terminal node belong to Group 3.

*Terminal node D3*. This group corresponds to non-technified farms (100.00%), with veterinary control in 54.55% of cases, and are not infested by ticks. All farms are resistant to amitraz, followed by alpha-cypermethrin in 63.64% of farms and ivermectin (36.36%). Regarding combined resistance, 63.64% of the farms showed resistance to both amitraz and alpha-cypermethrin. Their cost for acaricide drugs is 30.95% of the costs of veterinary inputs (691.19 USD per year). Most of the farms in this terminal node belong to Group 4.

*Terminal node E3*. This group corresponds to non-technified farms (81.82%), with veterinary control in 81.82% of cases, and with high levels of tick infestation (100.00%). Its main problem is resistance to amitraz in 54.55% of the farms, followed by alpha-cypermethrin (45.45%) and ivermectin (36.36%). Regarding combined resistance, 36.36% of the farms showed resistance to both amitraz and alpha-cypermethrin. Their cost for acaricide drugs is 48.60% of the costs of veterinary inputs, which were 568.37 USD on average per year. Most of the farms in this terminal node belong to Group 1.

*Terminal node F3*. This group corresponds to non-technified farms (64.29%), with veterinary control in 92.86% of cases, and with high levels of tick infestation (100.00%). The farms are resistant to amitraz in 35.71%, followed by ivermectin (28.57%). In addition, they are sensitive to alpha-cypermethrin. Regarding combined resistance, 21.43% of the farms showed resistance to both amitraz and ivermectin. Their cost for acaricide drugs is 30.74% with respect to the costs of veterinary inputs, on average 1286.04 USD per year. The farms belonging to this node are part of small (Group 1) and medium non-technified cattle farms (Group 3).

*Terminal node G3*. This group corresponds to non-technified farms (70.00%), with veterinary control in 60.00% of cases, and with high levels of tick infestation (100.00%). Their main problem is resistance to alpha-cypermethrin in all farms, followed by amitraz (60.00%) and ivermectin (40.00%). Regarding combined resistance, 60.00% of the farms showed resistance to both amitraz and alpha-cypermethrin. Their cost for acaricide drugs is 44.28% of the costs of veterinary inputs, which was on average 736.66 USD per year. Most of the farms in this terminal node belong to Group 2.

#### Impact of acaricide resistance and tick infestation on sanitary costs

**Fig 8:**
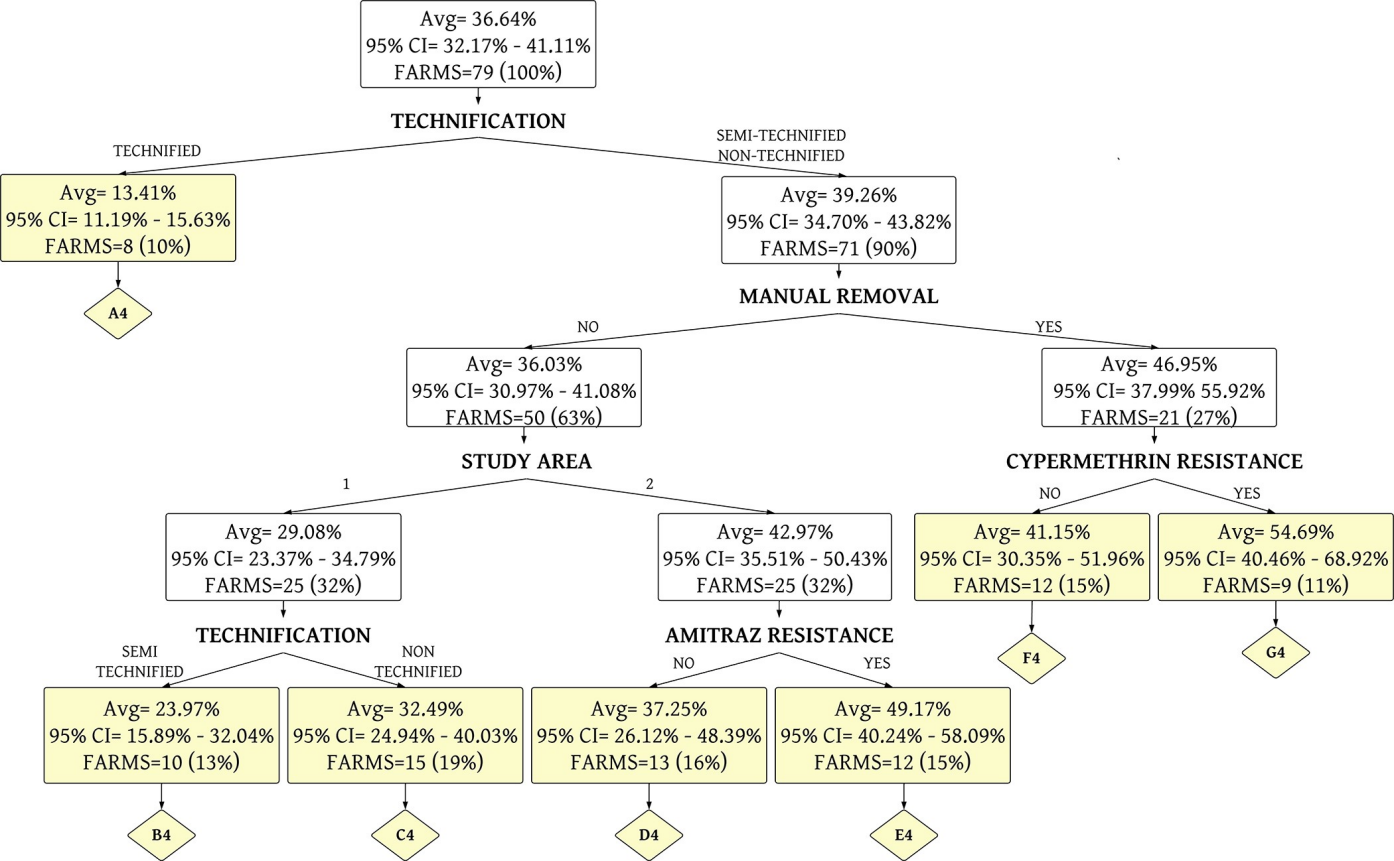
Decision tree analysis of the percentage of sanitary costs allocated to acaricide treatment (Model 4). A4 until G4 are the terminal nodes of the tree (yellow boxes). The average (Avg) corresponds to the % of sanitary costs destined for the acaricide treatment. CI is the confidence interval.

The percentage of acaricide treatment costs to management variables was grouped into seven homogeneous terminal nodes using a decision tree analysis (Fig 8 and S4 Table). This model presented a R^2^ of 0.37 and MSE of 15.91% in training data. The R^2^ was 0.38 and MSE was 16.80% in testing data.

The seven terminal nodes are described below.

*Terminal node A4*. This group corresponds to technified farms (100.00%), with veterinary control in 87.50% of cases; high level of tick infestation in 25.00% of farms. The main problem is resistance to amitraz in 75.00% of the farms, followed by alpha-cypermethrin (50.00%) and ivermectin (25.00%). Regarding combined resistance, 50.00% of the farms showed resistance to both amitraz and alpha-cypermethrin. Their cost of acaricide treatment is 13.41% of the 2975.83 USD on the average annual cost of veterinary inputs. Most of the farms in this terminal node belong to Group 5.

*Terminal node B4*. This group corresponds to semi-technified farms (100.00%), with veterinary control in all cases; a high level of tick infestation in 40.00% of farms. Its main problem is resistance to alpha-cypermethrin and ivermectin, with 70.00% in both cases, followed by amitraz with 60.00%. Regarding combined resistance, 60.00% of the farms showed resistance to amitraz, alpha-cypermethrin, and ivermectin. Their cost of acaricide treatment is 23.97% of the 934.49 USD on average annual of veterinary inputs. The farms belonging to this node are part of small (Group 1) and medium semi-technified cattle farms (Group 4).

*Terminal node C4*. This group corresponds to non-technified farms (100.00%), with veterinary control in 86.67% of cases; a high level of tick infestation in 66.67% of farms. Its main problem is resistance to alpha-cypermethrin with 53.33%, followed by amitraz (33.33%) and ivermectin (13.33%). Regarding combined resistance, 20.00% of the farms showed resistance to both amitraz and alpha-cypermethrin. Their cost of acaricide treatment is 32.49% of the 1251.32 USD on average annual of veterinary input costs. Most of the farms in this terminal node belong to Group 3.

*Terminal node D4*. This group corresponds to non-technified farms (61.54%), with veterinary control in 38.46% of cases; a high level of tick infestation in 46.15% of farms. These farms are sensitive to amitraz; its main problem is resistance to alpha-cypermethrin and ivermectin, with 38.46% in both cases. Regarding combined resistance, only 7.69% of the farms showed resistance to both alpha-cypermethrin and ivermectin. Their cost of acaricide treatment is 37.25% of the 2322.71 USD on average annual of veterinary input costs. Most of the farms in this terminal node belong to Group 3.

*Terminal node E4*. This group corresponds to non-technified farms (75.00%), with veterinary control in 25.00% of cases; a high level of tick infestation in 41.67% of farms. All farms are resistant to amitraz, followed by alpha-cypermethrin in 75.00% of farms and ivermectin (50.00%). Regarding combined resistance, 75.00% of the farms showed resistance to both amitraz and alpha-cypermethrin. Their cost of acaricide treatment is 49.17% of the 1331.13 USD on average annual of veterinary inputs. The farms belonging to this node are part of medium cattle farms (Groups 2, 3 and 4).

*Terminal node F4*. This group corresponds to non-technified farms (75.00%), with veterinary control in 91.67% of cases; a high level of ticks infestation in 50.00% of farms. The farms are resistant to amitraz in 41.67%, followed by ivermectin (3.33%). In addition, they are sensitive to alpha-cypermethrin. Regarding combined resistance only, 8.33% of the farms showed resistance to amitraz, alpha-cypermethrin, and ivermectin. Their cost of acaricide treatment is 41.15% of the 1563.37 USD on average annual of veterinary inputs. Most of the farms in this terminal node belong to Group 1.

*Terminal node G4*. This group corresponds to non-technified farms (77.78%), with veterinary control in 55.56% of cases; a high level of ticks infestation in 44.44% of farms. Its main problem is resistance to alpha-cypermethrin in all farms, followed by amitraz (55.56%) and ivermectin (33.33%). Regarding combined resistance, 55.56% of the farms showed resistance to both amitraz and alpha-cypermethrin. Their cost of acaricide treatment is 54.69% of the 917.42 USD on average annual of veterinary inputs. The farms belonging to this node are part of small (Group 1) and medium cattle farms (Groups 3 and 4).

## Discussion

### Tick infestation and acaricide resistance

Although the non-significant differences between the high level of tick infestation and tick-acaricide resistance, the decision trees indicated that farms without acaricide resistance had higher levels of tick infestation compared to those farms without resistance showing that this effect is more complex than the direct effect. This effect can be associated with the fact that for successful tick control requires, both the parasitic and non-parasitic phases must be controlled. The lack of control of the non-parasitic phase results in a high population of tick larvae remaining in the paddocks and rapidly reinfesting animals after chemical control [41, 42], regardless of whether the farm is resistant to acaricides or not. The classification model (Fig 2) presented an AUC = 0.64. The AUC is an important metric for classification, and it is often used as a measure of model performance. In our tree model, the level of technology had an important inverse relationship with the presence of high infestation and resistance to acaricides. Farms with higher technification are less infested (25.00%) than semi-technified and non-technified farms, as reported previously [28].

Resistance to acaricides results showed that 49.52% of farms had resistance to amitraz, 53.33% to alpha-cypermethrin, and 37.14% to ivermectin, respectively. Rodríguez-Hidalgo et al. (2017) [15] already reported resistance to these acaricides in 67.00 (amitraz), 50.00% (cypermethrin), and 25.00% (ivermectin) of the farms, using a similar methodology in another subtropical zone. The resistance levels are similar to those reported in this study. The amitraz resistance allele in R. microplus was also found in 62% of farms of other Ecuadorian zone (Santo Domingo de los Tsáchilas) indicating its widespread character [14].

Amitraz is considered the first line of action for tick control [43]. However, observing that its inefficacy, farmers opt for other acaricides applied in bath spray, such as alpha-cypermethrin or organophosphates. In Ecuador, alpha-cypermethrin and amitraz are marketed under different trade names and active ingredients (single or combined with other acaricides). This situation makes the farmer believe that he is rotating the active ingredient but, he is using the same one, which encourages increasing resistance. Also, it is well known that pyrethroids insecticides create a rapid evolution towards their [44, 45]. Similar situations occur with the use of ivermectin. Ivermectin and doramectin are the only injectable antiparasitic products marketed for the control of endo- and ectoparasites in cattle [46].

### Farm typology and economic analysis

Five groups of farms were formed, and they were classified as small farms (one group), medium farms (three groups), and large farms (one group). Similarly to other studies [47–49], labour force and supplementary feeding are the most important items used for the classification of farmers, representing 43.42% and 26.03% of the total costs, respectively.

While on large farms, the main item for the classification is the cost of supplemental feed, on small and medium farms, it is the labour force. In both groups, grazing is the main source of animal feed [50, 51]. In groups 1, 2, and 4, unpaid family labour accounts for more than 70% of the labour force, consistent with previous studies realised by Paez (2001) [52] and Posadas et al. (2014) [53], where family labour predominates in farms with low productive intensity. The use of family labour on these farms represents a strategy to take advantage of the human capital of the family nucleus [53], and this characteristic has been observed in groups outside the labour market and where it is difficult to find a paid job, either because of their age or because they could not be employed full-time [54].

As for the administration by women on the farms studied, their presence was determined, but there was no majority in any group. Female management occupies values close to 50% in Group 1 (47.52%) and Group 2 (47.06%) farms. However, it corresponds to small and medium farms that are not technified.

The costs of feeding are the main costs of livestock farming and are associated with farmers trying to make up for the nutrient deficiency of the pastures or low paddock availability, by using concentrates, silage, hay, and by-products (brewer’s bran, molasses). Moreover, farmers spend less than 1% of the costs in purchasing fertilisers or herbicides for the paddocks, so that they prefer to invest directly in the animals instead of the pastures. In addition, tropical and subtropical livestock farming in developing countries, and in Ecuador in particular, usually is developed in vulnerable areas, where soils have few nutrients and where sustaining pastures for a long time seems difficult [55].

The impact of veterinary inputs (E6) on these dairy production systems is approximately 8%, in agreement with Carmona and Vindas (2007) [56], where this cost ranged between 5–8%. This cost ranks third from the total costs, behind the cost of labour and supplementary feed. Rees et al. [57] and Tang et al. [58] mentioned that reducing the use of drugs in livestock contributes to reducing antimicrobial resistance in both human and animal populations. On the other hand, the cost of veterinary services is among what the farmer spends the least on (1.25%). If the opportunity cost (subsidised veterinary services) would be considered, it will increase to 1.99%. These two values show farmers’ low use of veterinary services, regardless of whether they pay for them or not. Romero and Villamil [59], found that in developing countries, most farmers rarely use veterinary services and rely first on their experience, followed by the farm worker or neighbours.

The cost of production per litre of milk (CPM) is 0.30 USD (95% CI: 0.17–0.43), similar to Hoyos et al. [49], where the weighted average was 0.27 USD. It should be emphasised that in our study, the CPM was determined by limiting the family labour force to the number of people needed to carry out livestock work. Other current studies conducted in the country indicate that the CPM is between 0.35 USD [60] and 0.43 USD [47]. However, these data vary widely depending on the area where the study was conducted and the size of the producers [49]. Within the groups, group 3 stands out as the most efficient. In this group, the average CPM of 0.25 USD is lower than the average cost of groups 4 and 5, which are more technified groups. In addition to this, group 3 produces 14,000 litres of milk more per year compared to group 4, which has a similar amount of land. We relate a higher CPM of group 4 to the fact that this group uses family labour in the management, which is not efficient and increases production costs, as labour is one of the main elements of livestock production. Although group 4 is semi-technified, most farms belong to the Quijos Valley Zone, where the parish government provides veterinary technical advice, free insemination, and in many cases, provides infrastructure; these parameters mean that this group is classified as semi-technified despite not having mechanical management in most cases. In addition, the location of most of the farms in group 3 in the northwest of Pichincha, where there is relatively better pasture quality due to the climate and soil quality, means that this group has a lower CPM and produces a higher quantity of milk than group 4. On the other hand, in group 5, despite having more land and technology, the average production cost is higher than in group 3, which was associated with the fact that although there is a large extension of land, farmers are not able to give good maintenance to the pastures [61], which decreases the efficiency in milk production.

Most farmers deliver their production to the local milk industry or to local cheese industry and receive on average 0.37 USD per litre for the raw milk. According to Ecuador’s Ministerial Resolution 394, the price per litre of milk corresponds to 52.4% of the retail price of UHT milk (0.80 USD) plus quality bonuses, which represents a remuneration near to 0.42 USD per litre of raw milk [62]. As observed in this and other studies [49, 60, 63], this base price is not met in most cases. Only group 5, large farms that deliver milk to the regional industry receive on average of 0.47 USD per litre of milk usually because of the farm location and better milk quality. In addition, the regional industries recognise and pay the bonuses established by the state for being certified brucellosis and tuberculosis free farms (0.01 USD per litre of raw milk) and certified farms with good livestock practices (0.02 USD per litre of raw milk) [62]. From the other hand, the low remuneration registered in this study may also be associated with the fact that the data was collected in 2020–2021, during the COVID-19 pandemic. Furthermore, according to our reports, 69.5% of farmers reported decreased income from milk sales due to the pandemic. As a result, milk collectors decreased the payment per litre of milk (indirect sales) or limited the volume of milk to be commercialised.

#### Impact of acaricide resistance and tick infestation on acaricide drug and treatment costs

When determining the percentage of veterinary inputs used for acaricide control (Model 3), it was observed that the semi-technified farms, despite not having animals highly infested animals by ticks, spent more than twice (25.34%) the costs of acaricide products compared to the technified farms, which spend less for this purpose (12.29%). Similar results are obtained in model 4, which analyses the acaricide drugs and labour used for acaricide treatment. Here, technified farms allocate a lower percentage of sanitary expenses (13.41%) to this activity compared to semi-technified (23.97%) and non-technified farms with (32.49%). In non-technified farms when observing the results of farms with and without resistance to amitraz in Model 3, farms without resistance (C3) spend a higher percentage of veterinary inputs (44.13%) in comparison with farms with resistance to amitraz (D3), which spend 30.95% of this cost. In could be due to the use of more expensive acaricides in those groups. When labour costs were included in Model 4 (as part of model prediction) farms with resistance (E4) spent a higher percentage 49.17% of the sanitary cost to control ticks in relation to farms without resistance to amitraz (37.25%), belonging to terminal node D4. In both models, it is observed that the farms corresponding to terminal nodes C3, D3 (Model 3) and D4, E4 (Model 4) share similar characteristics in terms of the level of low levels of technology and low level of tick infestation in animals. However, they differ in terms of resistance to other acaricides. Farms with higher acaricide expenditure (G3 and E4) are farms that have higher resistance to alpha-cypermethrin and ivermectin. This suggests that lower costs in tick control with acaricide is not only prevented by having sensitivity to amitraz; it is also the sum of good management use of alpha-cypermethrin and ivermectin; those farms, in most cases, did not have high level of tick infestation.

On the other hand, when analysing the terminal nodes of the farms with and without resistance to cypermethrin, the nodes are made up of farms with low or medium technification, and also, it was found that those farms have the highest percent of expenditure are those with resistance to alpha-cypermethrin (G3 and G4) in relation to those without resistance (F3 and F4). When analysing the typology of these farms, they have similarities in terms of the presence of high level of tick infestation (F3 and G3 = 100.00%; F4 and G4 = 50.00% and 44.44%, respectively) but differ in terms of veterinary control. Therefore, in these nodes, the farms with a lower percentage of expenditures for tick control are the farms with higher veterinary control. In the terminal nodes of Model 3, formed based on resistance or non-resistance to amitraz, farms with resistance to amitraz (C3) did not generate higher expenses than those without resistance. This did occur in farms with resistance to alpha-cypermethrin (F3). We associate this with the price difference of these acaricides. The use of alpha-cypermethrin accounts for the majority of expenditures on veterinary inputs, regardless of whether the farms are sensitive or not to amitraz. Acaricides, whose main active ingredient is alpha-cypermethrin, are generally marketed in association with other acaricides and also are applied as pour on animals and have a higher cost than amitraz-based drugs, which usually are not marketed in association with other ingredients. Cypermethrin’s are also seen by farmers as a rapid action product [64].

Model 1 shows that the cost of acaricide treatment varies according to the technification of the farms. Technified farms have a lower expenditure on acaricide treatment (1.30%) compared to semi-technified farms (3.43%) and non-technified farms (6.24%). Although resistance to alpha-cypermethrin plays a role in the formation of the decision tree, it does not affect the cost of the acaricide treatment if the farm has resistance. In addition to having or not resistance to alpha-cypermethrin, the cost of acaricide treatment varies according to the presence of high infestation and the degree of technification. Farms with high infestation spend more (4.28%) than farms without high infestation (2.74%). In addition, the labour invested to remove ticks manually increases the cost of on-farm acaricide treatment to 5.75%.

When analysing model 2, the average annual cost of acaricide treatment is 19.41 USD per adult animal per year. However, this cost increases or decreases like the other models depending on the size and level of technification. Large and technified farms (terminal node A2) have a treatment cost per animal of 8.46 USD, much lower than the general average. These farms have better access to the market and can negotiate prices by buying in volume, and are more efficient at applying acaricide treatments than the other groups. They use pour-on treatments (Fipronil and Fluazuron) and combination acaricides, which are more expensive. However, having fewer treatments per year does not increase the cost of treatment per year. At the other extreme, most small and non-technified farms are distributed in terminal nodes D2 and E2. These farms have a treatment cost per animal of 23.85 USD and 28.44 USD, respectively. About 42% of these farms have bad management of acaricides, mix different commercial presentations and overdose the recommended dose; they are also the groups with the highest number of spray baths per year. The higher cost of treatment on farms in terminal node E2 is associated with a higher percentage of farms with high infestation. In addition, many farms in this group (42.11%) mix commercial presentations, and the majority (89.00%) of the farms are located in the Valle de Los Quijos area, where they do not have the same access to agricultural warehouses where they can buy inputs. The medium-sized farms are distributed in terminal nodes B2 and C2 according to the presence or absence of resistance to alpha-cypermethrin. It is striking that this does not matter at the time of acaricide treatment, as the two groups use a similar percentage of alpha-cypermethrin-based acaricides, and the frequency of acaricide treatment is similar. Therefore, we associate the higher cost of animal treatment per year in terminal node B2 to the fact that there is greater use of treatments in the form of pour-on treatments and that these farms mix acaricides of different commercial presentations.

In general, the cost of annual acaricide treatment per animal varies according to the size and technology of the farms. Moreover, within groups with similar conditions, it varies according to the correct or incorrect use of acaricides (mixture of commercial presentations). Considering the treatment method, the cost of treatment increases on farms where pour-on or acaricides with a higher amount of active ingredients are used. There are several studies where the cost of acaricide treatment is calculated. For example, Frisch et al., in 2000 [65] determined that the cost per year of treating cattle on farms in northern Australia is 8.04 USD, which is close to that found on large, technified farms in our study. Other studies vary in price according to the type of acaricide used and the frequency of spray baths. For example, in Mexico [66], acaricide treatment with Ivermectin costs 53.23 USD, Organophosphates 8.23 USD and Cypermethrin 5.55 USD.

The typology formed did not fit exactly with the terminal nodes formed. It could be observed that there are very solid groups, such as group 5 of technified farms. However, the rest of the group is distributed in the terminal nodes formed by semi and non-technified farms, but not in a distinctive way, such as group 5 in terminal node 1.

## Conclusions

In conclusion, models 3 and 4 present similar results, in which we observe the importance of the level of farm technification, alpha-cypermethrin and amitraz resistance in terms of the percentage of money spent on acaricide control. In models 1 and 2, although alpha-cypermethrin resistance is part of the decision tree, when determining the annual cost of acaricide treatment at the farm and animal level, the presence of resistance did not increase the cost of treatment. It was found that in addition to having or not having resistance, the cost varies according to the level of infestation, technification and use of more expensive acaricides. In any model, did the presence or not of resistance to ivermectin play a major role, despite being an antiparasitic not only used to control ticks but also to control frequent internal parasites in the study areas such as *Paramphistomum* sp. and Fasciola hepatica [67, 68]. This observation is associated with the fact that dairy farmers are aware of the restriction of use in dairy cows and try not to use it or did not report its use in this study. Although the use of ivermectin did not have repercussions on the profitability of the farms, it is known that it can be public health from residues in milk and meat [69, 70]. The present study is one of the few studies describing the economic impact of diseases affecting production animals in Ecuador. Furthermore, it is the first to quantify the economic effects of tick presence. The data obtained in this research confirmed the impact of ticks on cattle farms in subtropical areas, especially on small and medium sized farms, which invested the most money in trying to control the presence of ticks. Therefore, in terms of research, it is recommended that studies be carried out to determine the extent of its use of ivermectin and to assess its putative health consequences on the population that consumes it. In term of decision-making, information campaigns using the present results should be developed in order to help farmers to improve the health status of their animals. Alternatively, the present results can contribute to the development of a control programme of tick infestation for small and medium-sized farms, which are the most affected in terms of the money they invest in controlling ticks.

## Supporting information

S1 Table Characterisation of the farms belonging to the terminal nodes of Model 1. The data correspond to the percentage of farms in each terminal node; Tech = Technified farms; semi = Semi-technified farms; non = Non technified farms; AM = farms with resistance to amitraz; CY = farms with resistance to alpha-cypermethrin; IV = farms with resistance to ivermectin; AM and CY = Farms with resistance to amitraz and alpha-cypermethrin; AM and IV = Farms with resistance to amitraz and ivermectin; CY and IV = Farms with resistance to alpha-cypermethrin and ivermectin; AM, CY and IV = Farms with resistance to amitraz, ivermectin, and alpha-cypermethrin.
(DOCX)

S2 Table Characterisation of the farms belonging to the terminal nodes of Model 2. The data correspond to the percentage of farms in each terminal node; Tech = Technified farms; semi = Semi-technified farms; non = Non technified farms; AM = amitraz; CY = alpha-cypermethrin; IV = ivermectin; ORG = Farms with resistance to amitraz and alpha-cypermethrin; FI = Fipronil (Fenilpirazoles); FLU = Fluazuron (Benzoylphenyl urea). * Buying commercial presentations with more than 2 active ingredients. ** Mixing of 2 or more commercial presentations with different or the same active ingredient.
(DOCX)

S3 Table Characterisation of the farms belonging to the terminal nodes of Model 3. The data correspond to the percentage of farms in each terminal node; Tech = Technified farms; semi = Semi-technified farms; non = Non technified farms; AM = farms with resistance to amitraz; CY = farms with resistance to alpha-cypermethrin; IV = farms with resistance to ivermectin; AM and CY = Farms with resistance to amitraz and alpha-cypermethrin; AM and IV = Farms with resistance to amitraz and ivermectin; CY and IV = Farms with resistance to alpha-cypermethrin and ivermectin; AM, CY and IV = Farms with resistance to amitraz, ivermectin, and alpha-cypermethrin.
(DOCX)

S4 Table Characterisation of the farms belonging to the terminal nodes of Model 4. The data correspond to the percentage of farms in each terminal node; Tech = Technified farms; semi = Semi-technified farms; non = Non technified farms; AM = farms with resistance to amitraz; CY = farms with resistance to alpha-cypermethrin; IV = farms with resistance to ivermectin; AM and CY = Farms with resistance to amitraz and alpha-cypermethrin; AM and IV = Farms with resistance to amitraz and ivermectin; CY and IV = Farms with resistance to alpha-cypermethrin and ivermectin; AM, CY and IV = Farms with resistance to amitraz, ivermectin, and alpha-cypermethrin.
(DOCX)
