# Supplementary material for: An economic evaluation of cattle tick acaricide-resistances and the financial losses in subtropical dairy farms of Ecuador: A farm system approach
Source: PLoS One. 2023 Jun 29;18(6):e0287104. doi: 10.1371/journal.pone.0287104 (PMC10309988; doi:10.1371/journal.pone.0287104)
Supplement: S1 Table — The data correspond to the percentage of farms in each terminal node; Tech = Technified farms; semi = Semi-technified farms; non = Non technified farms; AM = farms with resistance to amitraz; CY = farms with resistance to alpha-cypermethrin; IV = farms with resistance to ivermectin; AM and CY = Farms with resistance to amitraz and alpha-cypermethrin; AM and IV = Farms with resistance to amitraz and ivermectin; CY and IV = Farms with resistance to alpha-cypermethrin and ivermectin; AM, CY and IV = Farms with resistance to amitraz, ivermectin, and alpha-cypermethrin. (DOCX) [file pone.0287104.s001.docx]

**Table S1. Characterisation of the farms belonging to the terminal nodes of Model 1.**

| **Variable** | **Terminal nodes of model 1** | | | | | |
| --- | --- | --- | --- | --- | --- | --- |
|  | **A1** | **B1** | **C1** | **D1** | **E1** | **F1** |
| Veterinary control presence | 100.00 | 85.71 | 57.14 | 47.37 | 54.55 | 84.21 |
| Highly Infested Farms | 25.00 | 71.43 | 50.00 | 0.00 | 100.00 | 42.11 |
| Manual tick removal | 62.50 | 0.00 | 0.00 | 0.00 | 0.00 | 100.00 |
| Presence of external paddocks | 25.00 | 42.86 | 35.71 | 5.26 | 54.55 | 68.42 |
| Level of technification | tech | semi | non | non | non | non |
| AM Resistance | 50,00 | 14,29 | 42,86 | 57,89 | 54,55 | 52,63 |
| IV Resistance | 12,50 | 42,86 | 35,71 | 47,37 | 45,45 | 31,58 |
| CY Resistance | 37,50 | 0,00 | 0,00 | 100,00 | 100,00 | 42,11 |
| AM and CY Resistance | 37,50 | 0,00 | 0,00 | 57,89 | 54,55 | 26,32 |
| AM and IV Resistance | 12,50 | 14,29 | 14,29 | 36,84 | 18,18 | 21,05 |
| CY and IV Resistance | 12,50 | 0,00 | 0,00 | 47,37 | 45,45 | 15,79 |
| AM, CY and IV Resistance | 12,50 | 0.00 | 0,00 | 36,84 | 18.18 | 15.79 |
| Study area 1 | 62,50 | 57,14 | 57,14 | 42,11 | 63,64 | 68,42 |
| Study area 2 | 37,50 | 42,86 | 42,86 | 57,89 | 36,36 | 31,58 |
| Typology Group 1 | 12,50 | 14,29 | 14,29 | 5,26 | 27,27 | 42,11 |
| Typology Group 2 | 0,00 | 28,57 | 21,43 | 10,53 | 36,36 | 15,79 |
| Typology Group 3 | 0,00 | 14,29 | 42,86 | 36,84 | 18,18 | 10,53 |
| Typology Group 4 | 0,00 | 14,29 | 21,43 | 47,37 | 9,09 | 26,32 |
| Typology Group 5 | 87,50 | 28,57 | 0,00 | 0,00 | 9,09 | 5,26 |

The data correspond to the percentage of farms in each terminal node; Tech=Technified farms; semi=Semi-technified farms; non=Non technified farms; AM = farms with resistance to amitraz; CY = farms with resistance to alpha-cypermethrin; IV = farms with resistance to ivermectin; AM and CY= Farms with resistance to amitraz and alpha-cypermethrin; AM and IV= Farms with resistance to amitraz and ivermectin; CY and IV= Farms with resistance to alpha-cypermethrin and ivermectin; AM, CY and IV= Farms with resistance to amitraz, ivermectin, and alpha-cypermethrin.
