# Supplementary material for: An economic evaluation of cattle tick acaricide-resistances and the financial losses in subtropical dairy farms of Ecuador: A farm system approach
Source: PLoS One. 2023 Jun 29;18(6):e0287104. doi: 10.1371/journal.pone.0287104 (PMC10309988; doi:10.1371/journal.pone.0287104)
Supplement: S2 Table — The data correspond to the percentage of farms in each terminal node; Tech = Technified farms; semi = Semi-technified farms; non = Non technified farms; AM = amitraz; CY = alpha-cypermethrin; IV = ivermectin; ORG = Farms with resistance to amitraz and alpha-cypermethrin; FI = Fipronil (Fenilpirazoles); FLU = Fluazuron (Benzoylphenyl urea). * Buying commercial presentations with more than 2 active ingredients. ** Mixing of 2 or more commercial presentations with different or the same active ingredient. (DOCX) [file pone.0287104.s002.docx]

**Table S2. Characterisation of the farms belonging to the terminal nodes of Model 2.**

| Variable | Terminal nodes of model 2 | | | | |
| --- | --- | --- | --- | --- | --- |
|  | **A2** | **B2** | **C2** | **D2** | **E2** |
| Veterinary control presence | 87.50 | 42.86 | 52.17 | 65.29 | 84.21 |
| Highly Infested Farms | 37.50 | 57.14 | 52.17 | 35.71 | 52.63 |
| Manual tick removal | 50.00 | 0.00 | 0.00 | 100.00 | 52.63 |
| Presence of external paddocks | 12.50 | 35.71 | 26.09 | 78.57 | 57.89 |
| Level of technification | tech | non | non | non | non |
| AM Resistance | 75.00 | 35.71 | 47.83 | 50.00 | 57.89 |
| IV Resistance | 25.00 | 35.71 | 47.83 | 42.86 | 47.37 |
| CY Resistance | 62.50 | 0.00 | 100.00 | 57.14 | 47.37 |
| AM and CY Resistance | 62.50 | 0.00 | 47.83 | 42.86 | 36.84 |
| AM and IV Resistance | 25.00 | 14.29 | 26.09 | 28.57 | 31.58 |
| CY and IV Resistance | 25.00 | 0.00 | 47.83 | 28.57 | 26.32 |
| AM, CY and IV Resistance | 25.00 | 0.00 | 26.09 | 28.57 | 21.05 |
| Study area 1 | 25.00 | 35.71 | 39.13 | 42.86 | 89.47 |
| Study area 2 | 75.00 | 64.29 | 60.87 | 57.14 | 10.53 |
| Typology Group 1 | 0.00 | 14.29 | 8.70 | 42.86 | 42.11 |
| Typology Group 2 | 0.00 | 14.29 | 17.39 | 21.43 | 31.58 |
| Typology Group 3 | 0.00 | 50.00 | 39.13 | 21.43 | 0.00 |
| Typology Group 4 | 0.00 | 14.29 | 26.09 | 14.29 | 26.32 |
| Typology Group 5 | 100.00 | 7.14 | 8.70 | 0.00 | 0.00 |
| Use of acaricide treatment with AM | 25,00 | 42,86 | 52,17 | 78,57 | 63,16 |
| Use of acaricide treatment with CY | 50,00 | 64,29 | 60,87 | 64,29 | 89,47 |
| Use of acaricide treatment with ORG | 0,00 | 64,29 | 43,48 | 78,57 | 47,37 |
| Use of acaricide treatment with IV | 50,00 | 78,57 | 78,26 | 92,86 | 63,16 |
| Use of acaricide treatment with FI | 25,00 | 35,71 | 17,39 | 21,43 | 26,32 |
| Use of acaricide treatment with FLU | 25,00 | 35,71 | 8,70 | 14,29 | 5,26 |
| Use of pour-on acaricide | 50.00 | 64.29 | 26.09 | 35.71 | 26.32 |
| Frequency of spraying baths (Days) | 53.42 | 28.52 | 26.74 | 15.79 | 19.63 |
| Number of active ingredients (acaricides) used annually | 1.88 | 3.29 | 2.61 | 3.50 | 2.95 |
| Use of acaricides with more than 2 active ingredients^*^ | 50.00 | 57.14 | 60.87 | 71.43 | 84.21 |
| Incorrect dosage of acaricides | 12.50 | 71.43 | 65.22 | 42.86 | 42.11 |
| Subdoses | 0.00 | 14.29 | 8.70 | 7.4 | 5.26 |
| Overdose | 0.00 | 50.00 | 56.52 | 35.71 | 36.84 |
| Mixture of acaricides^**^ | 12.50 | 35.71 | 17.39 | 21.43 | 42.11 |

The data correspond to the percentage of farms in each terminal node; Tech=Technified farms; semi=Semi-technified farms; non=Non technified farms; AM =amitraz; CY = alpha-cypermethrin; IV = ivermectin; ORG= Farms with resistance to amitraz and alpha-cypermethrin; FI= Fipronil (Fenilpirazoles); FLU= Fluazuron (benzoylphenyl urea). ^*^ Buying commercial presentations with more than 2 active ingredients. ^**^ Mixing of 2 or more commercial presentations with different or the same active ingredient.
