# Supplementary material for: An economic evaluation of cattle tick acaricide-resistances and the financial losses in subtropical dairy farms of Ecuador: A farm system approach
Source: PLoS One. 2023 Jun 29;18(6):e0287104. doi: 10.1371/journal.pone.0287104 (PMC10309988; doi:10.1371/journal.pone.0287104)
Supplement: S3 Table — The data correspond to the percentage of farms in each terminal node; Tech = Technified farms; semi = Semi-technified farms; non = Non technified farms; AM = farms with resistance to amitraz; CY = farms with resistance to alpha-cypermethrin; IV = farms with resistance to ivermectin; AM and CY = Farms with resistance to amitraz and alpha-cypermethrin; AM and IV = Farms with resistance to amitraz and ivermectin; CY and IV = Farms with resistance to alpha-cypermethrin and ivermectin; AM, CY and IV = Farms with resistance to amitraz, ivermectin, and alpha-cypermethrin. (DOCX) [file pone.0287104.s003.docx]

**Table S3. Characterisation of the farms belonging to the terminal nodes of Model 3.**

| Variable | Terminal nodes of the model 3 | | | | | | |
| --- | --- | --- | --- | --- | --- | --- | --- |
|  | **A3** | **B3** | **C3** | **D3** | **E3** | **F3** | **G3** |
| Veterinary control presence | 90.00 | 72.73 | 41.67 | 54.55 | 81.82 | 92.86 | 60.00 |
| Highly Infested Farms | 20.00 | 0.00 | 0.00 | 0.00 | 100.00 | 100.00 | 100.00 |
| Manual tick removal | 70.00 | 27.27 | 25.00 | 45.45 | 100.00 | 0.00 | 0.00 |
| Presence of external paddocks | 30.00 | 36.36 | 16.67 | 9.09 | 72.73 | 57.14 | 60.00 |
| Level of technification | tech | semi | non | non | non | non | non |
| AM Resistance | 70.00 | 36.36 | 0.00 | 100.00 | 54.55 | 35.71 | 60.00 |
| IV Resistance | 20.00 | 36.36 | 50.00 | 36.36 | 36.36 | 28.57 | 40.00 |
| CY Resistance | 50.00 | 63.64 | 66.67 | 63.64 | 45.45 | 0.00 | 100.00 |
| AM and CY Resistance | 50.00 | 36.36 | 0.00 | 63.64 | 36.36 | 0.00 | 60.00 |
| AM and IV Resistance | 20.00 | 27.27 | 0.00 | 36.36 | 18.18 | 21.43 | 20.00 |
| CY and IV Resistance | 20.00 | 27.27 | 25.00 | 27.27 | 18.18 | 0.00 | 40.00 |
| AM, CY and IV Resistance | 20.00 | 27.27 | 0.00 | 27.27 | 18.18 | 0.00 | 20.00 |
| Study area 1 | 50.00 | 72.73 | 41.67 | 45.45 | 81.82 | 71.43 | 80.00 |
| Study area 2 | 50.00 | 27.27 | 58.33 | 54.55 | 18.18 | 28.57 | 20.00 |
| Typology Group 1 | 10.00 | 27.27 | 8.33 | 9.09 | 45.45 | 35.71 | 20.00 |
| Typology Group 2 | 0.00 | 9.09 | 25.00 | 18.18 | 18.18 | 14.29 | 50.00 |
| Typology Group 3 | 0.00 | 27.27 | 41.67 | 9.09 | 18.18 | 28.57 | 20.00 |
| Typology Group 4 | 0.00 | 36.36 | 25.00 | 63.64 | 18.18 | 14.29 | 10.00 |
| Typology Group 5 | 90 | 0.00 | 0.00 | 0.00 | 0.00 | 7.14 | 0.00 |

The data correspond to the percentage of farms in each terminal node; Tech=Technified farms; semi=Semi-technified farms; non=Non technified farms; AM = farms with resistance to amitraz; CY = farms with resistance to alpha-cypermethrin; IV = farms with resistance to ivermectin; AM and CY= Farms with resistance to amitraz and alpha-cypermethrin; AM and IV= Farms with resistance to amitraz and ivermectin; CY and IV= Farms with resistance to alpha-cypermethrin and ivermectin; AM, CY and IV= Farms with resistance to amitraz, ivermectin, and alpha-cypermethrin.

**Table S4. Characterisation of the farms belonging to the terminal nodes of Model 4.**

| Variable | Terminal nodes of model 4 | | | | | | |  |
| --- | --- | --- | --- | --- | --- | --- | --- | --- |
|  | **A4** | **B4** | **C4** | **D4** | **E4** | **F4** | **G4** | |
| Veterinary control presence | 87.50 | 100.00 | 86.67 | 38.46 | 25.00 | 91.67 | 55.56 | |
| Highly Infested Farms | 25.00 | 40.00 | 66.67 | 46.15 | 41.67 | 50.00 | 44.44 | |
| Manual tick removal | 75.00 | 0.00 | 0.00 | 0.00 | 0.00 | 100.00 | 100.00 | |
| Presence of external paddocks | 37.50 | 50.00 | 46.67 | 0.00 | 25.00 | 58.33 | 55.56 | |
| Level of technification | tech | semi | non | non | non | non | non | |
| AM Resistance | 75.00 | 60.00 | 33.33 | 0.00 | 100.00 | 41.67 | 55.56 | |
| IV Resistance | 25.00 | 70.00 | 13.33 | 38.46 | 50.00 | 3.33 | 33.33 | |
| CY Resistance | 50.00 | 70.00 | 53.33 | 38.46 | 75.00 | 0.00 | 100.00 | |
| AM and CY Resistance | 50.00 | 50.00 | 20.00 | 0.00 | 75.00 | 0.00 | 55.56 | |
| AM and IV Resistance | 25.00 | 50.00 | 6.67 | 0.00 | 50.00 | 8.33 | 33.33 | |
| CY and IV Resistance | 25.00 | 50.00 | 6.67 | 7.69 | 50.00 | 0.00 | 33.33 | |
| AM, CY and IV Resistance | 25.00 | 40.00 | 0.00 | 0.00 | 50.00 | 0.00 | 33.33 | |
| Study area 1 | 37.50 | 100.00 | 100.00 | 0.00 | 0.00 | 83.33 | 55.56 | |
| Study area 2 | 62.50 | 0.00 | 0.00 | 100.00 | 100.00 | 16.67 | 44.44 | |
| Typology Group 1 | 12.50 | 27.27 | 20.00 | 0.00 | 8.33 | 41.67 | 33.33 | |
| Typology Group 2 | 0.00 | 18.18 | 20.00 | 15.38 | 25.00 | 16.67 | 11.11 | |
| Typology Group 3 | 0.00 | 9.09 | 40.00 | 46.15 | 33.33 | 8.33 | 22.22 | |
| Typology Group 4 | 0.00 | 36.36 | 20.00 | 15.38 | 33.33 | 33.33 | 33.33 | |
| Typology Group 5 | 87.50 | 0.00 | 0.00 | 23.08 | 0.00 | 0.00 | 0.00 | |

The data correspond to the percentage of farms in each terminal node; Tech=Technified farms; semi=Semi-technified farms; non=Non technified farms; AM = farms with resistance to amitraz; CY = farms with resistance to alpha-cypermethrin; IV = farms with resistance to ivermectin; AM and CY= Farms with resistance to amitraz and alpha-cypermethrin; AM and IV= Farms with resistance to amitraz and ivermectin; CY and IV= Farms with resistance to alpha-cypermethrin and ivermectin; AM, CY and IV= Farms with resistance to amitraz, ivermectin, and alpha-cypermethrin.
